# Mitotic chromatin compaction tethers extrachromosomal DNA to chromosomes and prevents their mis-segregation into micronuclei

**DOI:** 10.1016/j.jbc.2025.111081

**Published:** 2025-12-20

**Authors:** Lu M. Yang

**Affiliations:** Department of Pathology, Stanford University School of Medicine, Stanford, California, USA

**Keywords:** extrachromosomal DNA, mitosis, chromatin compaction, HDAC, Ki67, micronuclei

## Abstract

Extrachromosomal DNA (ecDNA) is linked to aggressive cancer growth, treatment resistance, and shorter survival across a wide variety of cancers. ecDNA promotes intratumoral genetic heterogeneity, enhanced oncogene expression, and accelerated tumor evolution, driving tumor pathogenesis. ecDNA lacks centromeres and segregates to daughter cell nuclei during mitosis by tethering to chromosomes. However, the mechanisms involved in this tethering are incompletely understood. Here, I present evidence that ecDNA tethering to chromosomes is coupled with chromatin compaction during mitotic chromosome formation, which acts to generally increase chromatin-chromatin interaction. Using a cancer cell line model, I show that decompacting mitotic chromatin under hypotonic conditions and by increasing histone acetylation untethers ecDNA from chromosomes, leading to their mis-segregation into micronuclei after mitosis. Additionally, overexpression of the mitotic chromosome surfactant Ki67 untethers ecDNA from chromosomes, leading to their mis-segregation into micronuclei. These findings show that the mechanisms involved in chromatin compaction are important for tethering ecDNA to chromosomes and preventing their mis-segregation into micronuclei. I propose a model in which interactions between ecDNA chromatin fibers and chromosomal chromatin contribute to ecDNA segregation into daughter cells during cell division.

Focal amplifications involving proto-oncogenes are a hallmark of cancer (1, 2, 3). Focal amplifications can be located either on chromosomes, often forming homogeneously staining regions (HSRs), or on extrachromosomal DNA (ecDNA), traditionally known as double minutes (DMs) (4). ecDNA are chromatinized, large (ranging from 100s of kb to several Mb in size), circular, and acentric (5, 6, 7, 8, 9, 10, 11). Compared to HSRs and other forms of focal amplifications, ecDNA-containing cancers are associated with lower patient survival (12, 13). Importantly, ecDNA lack centromeres and tether to either sister chromatid during mitosis to ensure that they are segregated into daughter cell nuclei (9, 14, 15) (Fig. 1*A*). This stochastic manner of inheritance leads to unequal ecDNA segregation, thereby contributing to cancer heterogeneity (6, 16, 17, 18, 19, 20, 21). While first described in 1978, the mechanism underlying how ecDNA tether to mitotic chromosomes is only beginning to be elucidated (22). Recent studies suggest that mitotic ecDNA transcription (23, 24) has a role in mediating ecDNA tethering. I hypothesized that the molecular mechanisms that mediate the organization and compaction of mitotic chromosomes, acting as a general chromatin-attractive force to increase chromatin-chromatin interaction, also mediate ecDNA tethering.

**Figure 1:**
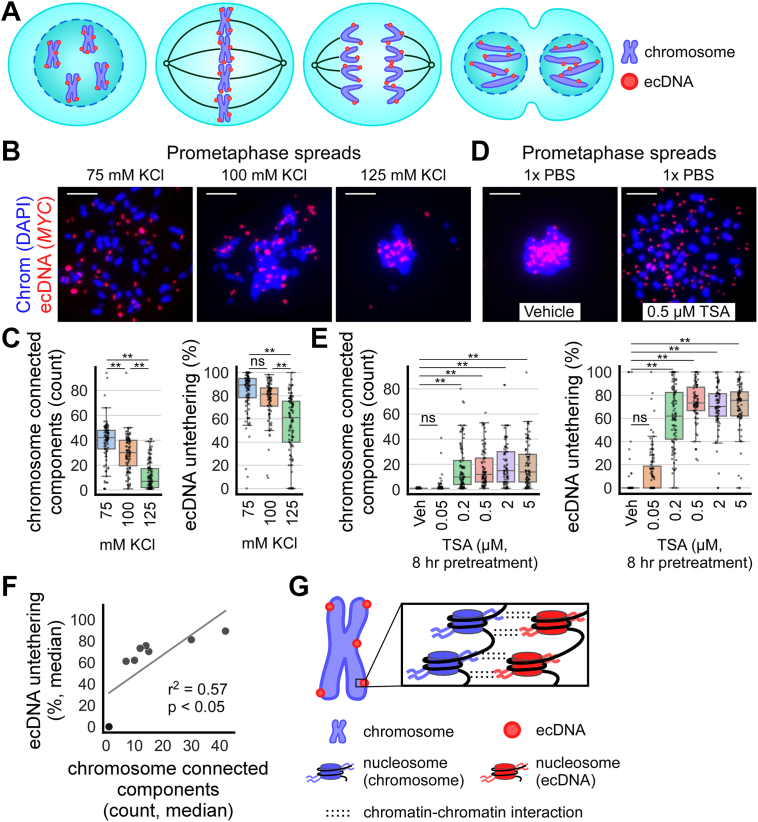
**The prometaphase spread technique untethers ecDNA and chromosomes by decompacting mitotic chromatin.***A*, schematic of an ecDNA-containing cell undergoing mitosis (from *left to right*: prophase, metaphase, anaphase, telophase). Acentric ecDNA (*red*) have been observed to tether to or “hitchhike” on chromosomes (*blue*) during mitosis to ensure their proper segregation and inheritance by daughter cells. *B*, prometaphase spreads performed on colcemid-arrested COLO320DM cells with the conventional hypotonic solution (75 mM KCl) and higher osmolarity solutions (100 mM and 125 mM KCl), producing varying amounts of chromosome (DAPI, *blue*) individualization and ecDNA (*MYC* FISH, *red*) untethering. *C*, quantification of *panel B*. *Left*: boxplots quantifying chromosome individualization in prometaphase spreads performed with varying solution osmolarity; from *left to right*, n = 3, 3, 3 biological replicates and 95, 92, 105 cells; one-way ANOVA, F = 100.7, *p* < 0.001; ∗∗*p* < 0.01 by Tukey’s honestly significant difference (HSD), ns = not significant. *Right*: quantification of ecDNA untethering; one-way ANOVA, F = 48.3, *p* < 0.001. Chromosome individualization is represented by the number of connected components identified as chromosomes by ecSeg. ecDNA untethering is represented by the number of ecDNA unattached to chromosomes divided by the total number of ecDNA not completely surrounded by chromosomes. *D*, prometaphase spreads performed with incubation in 1× PBS using COLO320DM cells pretreated for 8 h with vehicle (0.1% DMSO) or varying concentrations of Trichostatin A (TSA). *E*, quantification of *panel D*. *Left*: quantification of chromosome individualization in prometaphase spreads performed with 1× PBS on cells pretreated with TSA; n = 3, 3, 3, 3, 3, 3 biological replicates and 88, 113, 96, 80, 76, 84 cells; one-way ANOVA, F = 38.5, *p* < 0.001; ∗∗*p* < 0.01 by Tukey’s HSD, ns = not significant. *Right*: quantification of ecDNA untethering; one-way ANOVA, F = 166.5, *p* < 0.001. *F*, linear regression analysis of median chromosome individualization *versus* ecDNA untethering of prometaphase spread conditions in *panels B*–*E* (n = 9). *G*, schematic: electrostatic/hydrophobic mitotic compaction forces at the level of nucleosomes tether ecDNA (*red*) to mitotic chromosomes (*blue*). In *panels B–E*, scale bar = 10 μm and each data point in graphs represents one cell.

During cell division, mitotic spindles associate with chromosomes at centromeres to direct chromosomal segregation into daughter cell nuclei. Chromosomes form during mitosis and move along the mitotic spindles, ensuring the segregation of one genome copy into each of the daughter cell (25). They are discrete, cylindrical structures composed of densely packed chromatin, organized through a series of coordinated molecular mechanisms, including (1) topological organization of chromatin into loops with helical periodicity; (2) global chromatin compaction; and (3) the regulation of chromosome surface by surfactants such as Ki67 (25). *Mechanism 1:* the typical chromosome cylindrical form arises from chromatin loops organized around the chromosome axis by molecular motors such as condensin that mediate DNA loop extrusion (26, 27, 28, 29, 30, 31). These molecular motors help stiffen chromosomes (32, 33, 34, 35) and shape their mitotic structure (36, 37, 38, 39, 40). *Mechanism 2:* global chromatin compaction is driven by a network of dynamic and weak interactions between nucleosomes and chromatin-associated proteins that reduces chromatin volume (25). This process consists in the folding and local compaction of the 10 nm fiber of nucleosomes, giving rise to denser and irregular chromatin structure. Such global chromatin compaction is distinct from topological organization of mitotic chromosomes by condensin and other molecular motors (25, 39, 41, 42) and is regulated by histone deacetylation and free intracellular Mg^2+^ concentration that modulate chromatin-chromatin affinity interactions during mitosis (43, 44, 45, 46, 47). *Mechanism 3:* factors that regulate the chromosome surface mediate the spatial separation of chromatin into distinct chromosome bodies in late prophase, which prevents chromosome coalescence and enables spindle microtubules to access the kinetochores (25). The protein Ki67 has been shown to act as a chromosome surfactant by forming a repulsive layer of proteins and RNA at the periphery of mitotic chromosomes, thereby promoting chromosome individualization (48, 49, 50, 51).

Here, I use an ecDNA-containing cancer cell line to study the role of mitotic chromatin compaction on ecDNA tethering to chromosomes and their mitotic inheritance. I focus on disrupting mechanisms 2 and 3 *via* small molecule drug treatment, cell osmolarity alteration, and genetic manipulation. I show that mitotic chromatin decompaction, induced either by hypotonic conditions or histone deacetylase (HDAC) inhibition, disrupts ecDNA-chromosome tethering. Additionally, I identify Ki67, which coats the surface of both mitotic chromosomes (48, 49, 50, 51) and ecDNA, as a modulator of ecDNA tethering. Untethered ecDNAs have an increased likelihood of mis-segregating into micronuclei. I propose that global chromatin compaction, driven by increased chromatin-chromatin interactions (mechanism 2), contributes to ecDNA tethering by promoting chromatin interactions between ecDNA and chromosome. Furthermore, I propose that the surfactant properties of Ki67 (mechanism 3) control ecDNA tethering by coating the surface of both ecDNA and chromosomes.

## Results

### The prometaphase spread technique untethers ecDNA and chromosomes by decompacting mitotic chromatin

COLO320DM cells are a colorectal cancer cell line containing *MYC* amplification on ecDNA, which can be visualized using *MYC* fluorescence *in situ* hybridization (FISH). On prometaphase spreads of these cells, ecDNA appear spatially dissociated from the chromosomes, suggesting that they are untethered (example: Fig. 1*B*, leftmost panel). This untethering has also been observed in prometaphase spreads of other cell lines containing ecDNA (6). I reasoned that ecDNA untethering could be linked to the reduction of intracellular ion concentration caused by incubation in hypotonic solution during prometaphase spread preparations (Fig. S1*A*), which may disrupt interactions involved in mitotic chromatin compaction (52, 53, 54, 55).

To test this, I performed prometaphase spreads using higher osmolarity variations (100 mM and 125 mM KCl) of the conventional 75 mM KCl hypotonic solution (Fig. 1*B*). 100 mM and 125 mM KCl are still hypotonic relative to the cells, but the net inflow of water into cells is expected to be decreased compared to 75 mM KCl. Both higher osmolarity treatments significantly reduced chromosome individualization, as measured by the number of chromosome connected components detected using ecSeg (56), from 39.2 ± 16.8 (mean ± standard deviation) with 75 mM KCl to 29.3 ± 14.6 and 11.4 ± 11.0 with 100 mM and 125 mM KCl, respectively. Similarly, ecDNA untethering decreased with the higher osmolarity treatments, from 83.3% ± 18.6 with 75 mM KCl to 76.7% ± 16.9 and 55.4% ± 25.8 with 100 mM and 125 mM KCl, respectively (Fig. 1, *B* and *C*). These observations suggest that ionic strength modulates ecDNA tethering to chromosomes, possibly *via* decompacting mitotic chromatin.

In addition to ionic strength, chromatin compaction is modulated by histone tail acetylation (47, 55, 57). To assess whether acetylation modulates chromosome individualization and ecDNA untethering similarly to ionic strength, I treated cells with Trichostatin A (TSA), a pan-histone deacetylase (HDAC) inhibitor that causes histone hyperacetylation and decompacts mitotic chromatin (42, 44, 45, 46, 58). I treated COLO320DM cells for 8 h with varying concentrations of TSA before performing prometaphase spreads in 1× PBS rather than 75 mM KCl (Fig. 1*D*). Staining for pan-Histone H3 acetylation confirmed an increase in acetylation on both ecDNA and chromosomes in cells treated with TSA in a dose-dependent manner (Fig. S1, *B* and *C*). TSA-treated cells presented both increased chromosome individualization (17.3 ± 14.9 in 0.5 μM TSA-treated cells) and ecDNA untethering (73.5% ± 17.3 in 0.5 μM TSA-treated cells) compared to vehicle-treated cells (1.0 ± 0.2 chromosome individualization and 7.2% ± 22.8 ecDNA untethering) (Fig. 1, *D* and *E*). This indicates that histone hyperacetylation disrupts ecDNA tethering to chromosomes, possibly *via* decompacting mitotic chromatin.

Notably, under hypotonic conditions and in TSA-treated cells, ecDNA untethering correlates with chromosome individualization in prometaphase spreads (Fig. 1*F*), suggesting that both chromosome-chromosome and ecDNA-chromosome tethering are regulated by ionic strength and histone acetylation (Fig. 1*G*). These observations imply that chromosome individualization and ecDNA tethering may be governed by the interactions involved in chromatin compaction.

### Hypotonic conditions impair ecDNA tethering

**Figure 2:**
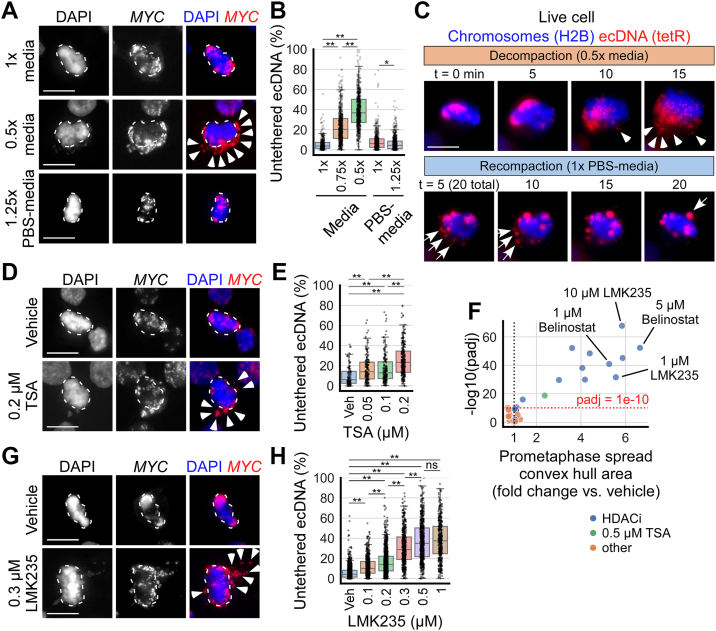
**Hypotonic conditions and HDAC inhibition untether ecDNA.***A*, fixed metaphase COLO320DM cells cultured on glass coverslips treated for 15 min with 1× media, 0.75× media (not shown), 0.5× media, 1:1 mix of 1× PBS with 1× media (1× PBS-media, not shown), and 1:1 mix of 1.5× PBS with 1× media (1.25× PBS-media); dashed outlines indicate metaphase plate chromosomes (DAPI, *blue*), arrowheads indicate untethered ecDNA (identified by *MYC* FISH signal, *red*). *B*, boxplots quantifying untethered ecDNA per cell from *panel A*, represented by % of *MYC* FISH signal in a cell unattached to chromosomes aligned at the metaphase plate (non-overlapping pixels); from left to right, n = 4, 6, 6, 7, 4 biological replicates and 427, 672, 691, 532, 686 cells; one-way ANOVA, F = 882.3, *p* < 0.001; ∗*p* < 0.05, ∗∗*p* < 0.01 by Tukey’s HSD. *C*, live imaging of COLO320DM cells expressing H2B-emiRFP670 (chromatin, *blue*) and tetR-mNeonGreen which binds to a tetO-96mer repeat sequence inserted near *MYC* loci (*MYC*, *red*). Cells were arrested at metaphase with 10 μM MG132 and placed in hypotonic 0.5× media at t = 0 min to decompact chromatin; arrows indicate untethered ecDNA; after image acquisition at t = 15 min, cells were placed in relatively normotonic 1× PBS-media to recompact chromatin; arrows indicate ecDNA-ecDNA tethering. Representative images of n = 5 biological replicates and >50 cells. *D*, fixed metaphase COLO320DM cells cultured on glass coverslips treated for 8 h with vehicle (0.1% DMSO) or indicated concentrations of TSA; dashed outlines indicate metaphase plate chromosomes (DAPI, *blue*), arrowheads indicate untethered ecDNA (identified by *MYC* FISH signal, *red*). *E*, boxplots quantifying untethered ecDNA per cell from *panel D*; from *left* to *right*, n = 3, 3, 3, 3 biological replicates and 98, 144, 161, 246 cells; one-way ANOVA, F = 34.9, *p* < 0.001; ∗∗*p* < 0.01 by Tukey’s HSD. *F*, modified volcano plot summarizing the results of the targeted screen for drugs with the ability to untether ecDNA and chromosomes, quantified by the area of the convex hull of the prometaphase spread produced without hypotonic solution incubation. TSA (0.5 μM) was included as positive control. padj = adjusted two-tailed Student’s *t* test *p* value using Bonferroni multiple test correction (44 total comparisons were made). Each data point represents the average of all cells for each condition. *G*, metaphase COLO320DM cells cultured on glass coverslips treated for 24 h with vehicle (0.1% DMSO) or indicated concentrations of LMK235; dashed outlines indicate metaphase plate chromosomes, arrowheads indicate untethered ecDNA. *H*, quantification of untethered ecDNA per cell from *panel* G; n = 11, 3, 7, 4, 3, 3 biological replicates and 1278, 501, 875, 472, 571, 388 cells; one-way ANOVA, F = 735.1, *p* < 0.001; ∗∗*p* < 0.01 by Tukey’s HSD, ns = not significant. In all panels, scale bar = 10 μm. Except in *panel F*, each data point in graphs represents one cell.

While prometaphase spreads allow visualization of separated chromosomes, they can alter nuclear organization. To further assess the link between chromatin compaction and ecDNA tethering in a more physiologically relevant context, I evaluated ecDNA tethering in COLO320DM metaphase cells cultured and fixed on glass coverslips. In these preparations, chromosomes are aligned at the metaphase plate, allowing the visualization of untethered ecDNA that detach from the metaphase plate while better preserving spatial relationships within the cell. To test the effects of variation in ionic conditions on ecDNA tethering, I treated cells for 15 min with solutions at five different ionic strengths: i. 1× media (isotonic control), ii. 0.75× media (hypotonic), iii. 0.5× media (hypotonic), iv. 1:1 mix of 1× PBS with 1× media (1× PBS-media, relatively isotonic control), and v. 1:1 mix of 1.5× PBS with 1× media (1.25× PBS-media, hypertonic). After treatment, cells were fixed for 10 min in paraformaldehyde dissolved in 1×, 0.75×, 0.5×, or 1.25× PBS matching the osmolarity of the incubation media to preserve the ionic conditions of the treatment. Cells were stained with DAPI to visualize DNA and *MYC* FISH to visualize ecDNA. ecDNA untethering significantly increased in cells incubated in hypotonic 0.75× (22.7% ± 15.2 untethered, mean ± standard deviation) and 0.5× (38.9% ± 17.1) media compared to 1× media (5.5% ± 6.0) (Fig. 2, *A* and *B*). Conversely, ecDNA untethering slightly but statistically significantly decreased in cells incubated in hypertonic 1.25× PBS-media (6.3% ± 6.8) compared to 1× PBS-media (8.3% ± 9.2). The effects of ionic strength on ecDNA tethering are consistent with known effects of ionic strength on chromatin compaction: Low salt conditions induce chromatin decompaction by reducing molecular interactions, while higher salt concentrations promote compaction (52, 53, 54, 55).

Prior studies have suggested that DNA double-strand breaks (DSBs) may cause ecDNA untethering, aggregation, and mis-segregation (59, 60, 61). To determine whether hypotonic and hypertonic treatments alter DSB levels, I examined by IF the expression of histone H2A.X phosphorylation (pH2A.X), a marker of DSB (62) which peaks at 2 to 3 min after DSB formation (63, 64). I found that while 0.5× hypotonic media incubation slightly decreased the number of pH2A.X foci per metaphase cell (Fig. S2, *A* and *B*), in every condition, the number of pH2A.X foci did not correlate with the extent of ecDNA untethering (Fig. S2*C*). This suggests that incubation in hypotonic media does not induce ecDNA untethering by promoting DSBs.

Next, I reasoned that mitotic chromatin in a hypercompacted state will be more resistant to ecDNA untethering by hypotonic incubation if the mechanism of untethering is *via* chromatin decompaction. Treatment of mitotic cells with 10 mM sodium azide (NaN_3_) and 50 mM 2-Deoxy-D-glucose (2-DG) has been shown to hypercompact chromatin by increasing the intracellular free Mg^2+^ concentration available to associate with chromatin (43). I treated cells for 3 h with 10 mM NaN_3_ + 50 mM 2-DG dissolved directly into the cell culture media, followed by a 15 min incubation in 1×, 0.75×, or 0.5× media. Chromatin hypercompaction by NaN_3_ + 2-DG significantly reduced the amount of ecDNA untethering by hypotonic 0.75× and 0.5× media (Fig. S2, *D* and *E*). Altogether, these results show that intracellular ionic strength regulates ecDNA tethering by modulating chromatin compaction.

To examine ecDNA untethering in real time, I employed live cell imaging using a COLO320DM cell line genetically modified to express histone H2B-emiRFP670 for chromatin visualization, and tetR-mNeonGreen that binds to a Tet-operator (tetO) array inserted onto ecDNA for ecDNA visualization (65). Cells were arrested at metaphase with MG132 (66) and placed in hypotonic 0.5× media (Fig. 2*C*). After 5 to 10 min of incubation in 0.5× media, ecDNA began moving away from the chromosomes aligned at the metaphase plate. At 15 min, most of the ecDNA appeared as smaller scattered foci, suggesting they have untethered from chromosomes and each other. To test for the reversibility of the untethering process, after 15 min in hypotonic 0.5× media, cells were placed back into isotonic conditions (1× PBS-media). I observed ecDNA compacting into larger foci, and by 20 min in isotonic conditions most of the ecDNA appeared retethered to the chromosomes or with each other (Fig. 2*C*). These results suggest that tethering and untethering are reversible processes, like chromatin compaction regulation by intracellular ionic strength (54).

### HDAC inhibition impairs ecDNA tethering

As described above, I found that treatment with the HDAC inhibitor TSA leads to untethering of ecDNA from chromosomes in prometaphase spreads. I confirmed this finding using COLO320DM metaphase cells cultured and fixed on glass coverslips, which, when treated with varying concentrations of TSA for 8 h, showed increased ecDNA untethering compared to vehicle-treated cells (9.9% ± 10.0 untethered, mean ± standard deviation): 17.2% ± 13.2 untethered with 0.05 μM TSA, 17.1% ± 14.4 with 0.1 μM, and 25.9% ± 15.7 with 0.2 μM; above 0.2 μM, I was unable to find many cells that progressed to metaphase (Fig. 2, *D* and *E*).

TSA is a broad-spectrum HDAC inhibitor blocking class I and II HDACs. To determine whether other HDAC inhibitors may similarly regulate ecDNA tethering to chromosomes, I performed a targeted screen of general and class-specific HDAC inhibitors (HDACi). 12 HDACi were tested, including Belinostat and Abexinostat (pan-HDACi (67)), Fimepinostat (HDAC class I and IIb inhibitor), Pracinostat (HDAC class I and II inhibitor), Mocetinostat and Entinostat (HDAC class I inhibitor), LMK235 and TMP269 (HDAC class IIa inhibitor), BRD73954 (HDAC class IIb inhibitor), Tasquinimod (HDAC4 inhibitor), RGFP966 (HDAC3 inhibitor (68)), and Santacruzamate A (HDAC2 inhibitor (68)). Additionally, I included nine non-HDACs inhibitor drugs with targets suggested to be involved in chromatin compaction through different mechanisms, such as Nicotinamide (pan sirtuin inhibitor (68)), EX527 (SIRT1 inhibitor (68)), CTPB (p300/CBP histone acetyltransferase activator (68, 69)), RRx001 (DNA methyltransferase inhibitor (70)), 5-Azacytidine (DNA methyltransferase inhibitor (71)), GSK343 (EZH2 inhibitor (71)), ZM447439 and Hesperadin (Aurora kinase inhibitor (72, 73)), and Paprotrain (MKLP-2 inhibitor (74)).

I treated COLO320DM cells with each of these drugs for 24 h at multiple concentrations ranges and performed prometaphase spreads using incubation in 1× PBS rather than the conventional hypotonic solution. I then quantified the area of the convex hull of prometaphase spreads (Fig. S2*F*) as an approximation of ecDNA and chromosome untethering. I found that only inhibitors of HDAC classes I and II effectively untethered ecDNA and individualized chromosomes, including Belinostat, Abexinostat, Fimepinostat, Pracinostat, and LMK235 (Figs. 2*F* and S2*G*). These effects were confirmed by treating COLO320DM cells cultured on glass coverslips with LMK235 or Belinostat for 24 h, which untethered ecDNA in a dose-dependent manner (Figs. 2, *G* and *H* and S2, *H* and *I*). I investigated the possibility of DSB formation as a confounding variable by examining pH2A.X IF and observed no correlation between the number of pH2A.X foci and the extent of ecDNA untethering (Fig. S2, *J*–*M*). This suggests that it is unlikely HDAC inhibition untethers ecDNA *via* inducing DSBs. Together, these findings suggest that HDAC inhibition untethers ecDNA from chromosomes by decompacting mitotic chromatin.

### Hypotonic conditions and HDAC inhibition lead to mis-segregation of ecDNA into micronuclei

**Figure 3:**
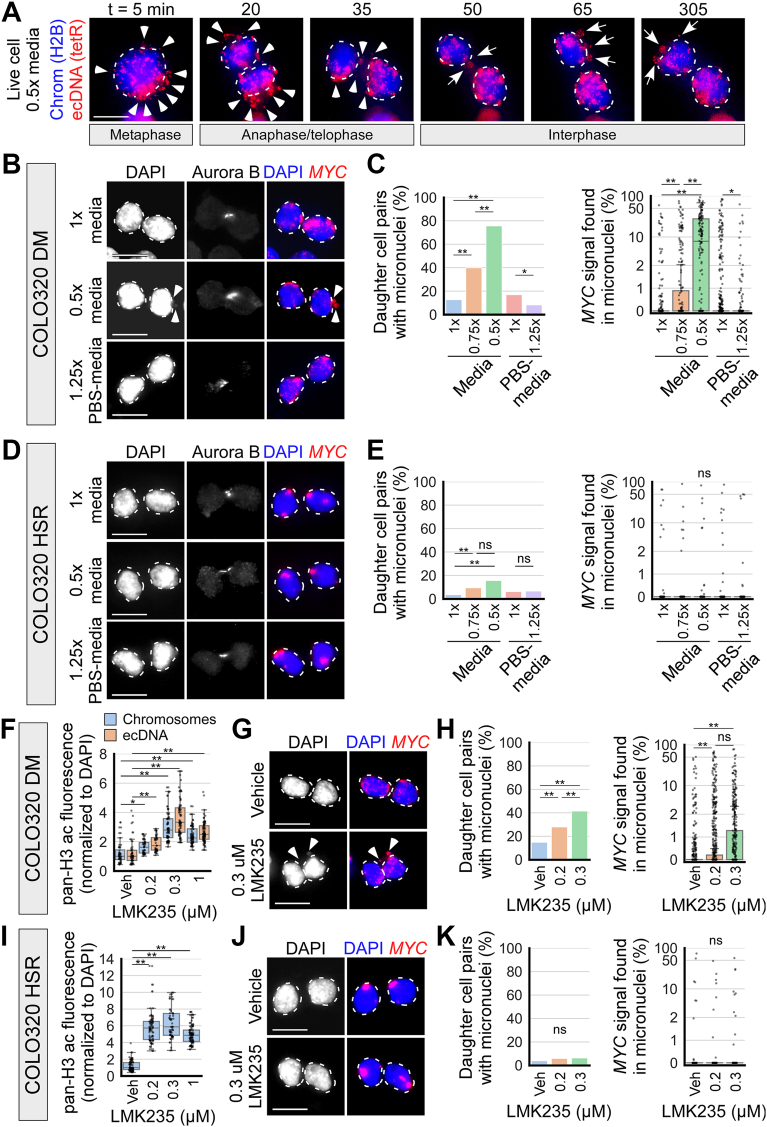
**Hypotonic conditions and HDAC inhibition lead to mis-segregation of ecDNA into micronuclei.***A*, live cell imaging of COLO320DM cells undergoing mitosis (chromatin labeled by H2B-emiRFP670, *blue*) and (*MYC* loci labeled by tetR-mNeonGreen, *red*). Mitotic cells were placed in 0.5× media at t = 0 min; *dashed outlines* indicate chromosomes/primary nuclei, arrowheads indicate untethered ecDNA, *arrows* indicate micronuclei. Representative image of n = 5 biological replicates and >50 cells. *B*, fixed newly-divided daughter COLO320DM cells, as identified by the presence of Aurora B staining, treated for 6 h with 1× media, 0.75× media (not shown), 0.5× media, 1× PBS-media (not shown), and 1.25× PBS-media; *dashed outlines* indicate primary nuclei, *arrowheads* indicate micronuclei. *C*, quantification of *panel B*. *Left*: quantification of the percentage of daughter cell pairs with micronuclei; chi-squared, ∗*p* < 0.05, ∗∗*p* < 0.01. *Right*: quantification of the percentage of all *MYC* FISH signal per daughter cell pair inside micronuclei as opposed to the primary nuclei (symlog scale, linear ≤2, log >2); one-way ANOVA, F = 151.4, *p* < 0.001, ∗*p* < 0.05, ∗∗*p* < 0.01; n = 3, 3, 3, 6, 4 biological replicates and 601, 233, 148, 881, 836 daughter cell pairs. *D* and *E*, fixed newly-divided daughter COLO320HSR cells, treated similarly as in *panels B* and *C*. *E*, *left*: chi-squared, ∗∗*p* < 0.01, ns = not significant. *Right*: one-way ANOVA, F = 0.9, *p* = 0.49; n = 3, 3, 3, 5, 3 biological replicates and 680, 256, 179, 578, 487 daughter cell pairs. *F*, quantification of pan-Histone H3 acetylation (pan-H3ac) IF in cytospin preparations of COLO320DM prometaphase spreads with chromosome and ecDNA segmentation by ecSeg (see Fig. S3*C*); cells were treated in vehicle or LMK235 for 24 h; n = 2, 2, 2, 2 biological replicates and 52, 44, 75, 84 cells; one-way ANOVA, chromosomes: F = 67.6, *p* < 0.001, ecDNA: F = 81.3, *p* < 0.001, ∗*p* < 0.05, ∗∗*p* < 0.01 by Tukey’s HSD. *G*, newly-divided daughter COLO320DM cells, as identified by the presence of Aurora B staining (not shown), treated for 24 h with vehicle (0.1% DMSO) or indicated concentrations of LMK235; *dashed outlines* indicate primary nuclei, *arrowheads* indicate micronuclei. *H*, quantification of *panel G*. *Left*: quantification of the percentage of daughter cell pairs with micronuclei; chi-squared, ∗∗*p* < 0.01. *Right*: quantification of the percentage of all *MYC* FISH signal per daughter cell pair inside micronuclei (symlog scale, linear ≤2, log >2); one-way ANOVA, F = 151.4, *p* < 0.001, ∗*p* < 0.05, ∗∗*p* < 0.01; n = 5, 5, 4 biological replicates and 713, 683, 420 daughter cell pairs. *I*, same as *panel F*, for COLO320HSR cells (chromosomes only; see Fig. S3*E*); n = 2, 2, 2, 2 biological replicates and 85, 85, 63, 99 cells; one-way ANOVA, F = 204.1, *p* < 0.001, ∗∗*p* < 0.01 by Tukey’s HSD. *J* and *K*, newly-divided daughter COLO320HSR cells, treated similarly as in *panels* G and H. *K*, *left*: chi-squared. *Right*: one-way ANOVA, F = 0.1, *p* = 0.88; n = 4, 3, 3 biological replicates and 486, 383, 335 daughter cell pairs. In all panels, scale bar = 10 μm and each data point in graphs represents one cell or daughter cell pair.

Next, to determine the fate of untethered ecDNA after cells exit mitosis, I live-imaged COLO320DM cells across mitoses in hypotonic 0.5× media (Fig. 3*A*). ecDNA untethering was observed throughout mitosis. Upon completion of mitosis, some of the untethered ecDNA were incorporated into the primary nuclei of the daughter cells, while some were in the cytoplasm, inside presumably micronuclei (Fig. 3*A*). This suggests that like lagging chromosomes and acentric chromatin fragments (75, 76), untethered ecDNA are likely to be mis-segregated into micronuclei.

To quantify the incorporation of untethered ecDNA into micronuclei, I examined micronuclei formation in fixed cells. I treated COLO320DM cells with 1× media, 0.75× media, 0.5× media, 1× PBS-media, and 1.25× PBS-media for 6 h to allow cells time to undergo mitosis under these conditions. I then fixed and identified newly divided daughter cells based on the presence of Aurora B kinase IF signal between the two daughters, as described previously (16) (Fig. 3*B*). I found that the percentage of daughter cell pairs containing micronuclei dramatically increased in cells incubated in hypotonic 0.75× (39.9%) and 0.5× (75.7%) media compared to 1× media (12.6%) (Fig. 3, *B* and *C*). In addition, the percentage of ecDNA (*MYC* FISH signal) within daughter cell pairs found inside micronuclei as opposed to the primary nuclei significantly increased in cells incubated in hypotonic 0.75× (4.2% ± 11.3, mean ± standard deviation) and 0.5× (16.5% ± 21.0) media compared to 1× media (0.6% ± 4.2) (Fig. 3*C*). Furthermore, the micronuclear contents were mainly ecDNA, as measured by the percentage of DAPI signal inside micronuclei that overlapped with *MYC* FISH signal (Fig. S3*A*). On the contrary, hypertonic treatment in 1.25× PBS-media decreased ecDNA incorporation into micronuclei compared to 1× PBS-media (Figs. 3, *B* and *C* and S3*A*).

To assess whether hypotonic conditions may induce micronuclei formation in cells that do not contain ecDNA, I compared *MYC* FISH signal incorporation into micronuclei between COLO320DM cells, in which *MYC* is amplified on ecDNA, and COLO320HSR cells, which are derived from the same parental cell line as COLO320DM cells, but in which *MYC* is amplified on chromosomes as homogeneously staining regions (HSR). The percentage of daughter cell pairs containing micronuclei increased in COLO320HSR cells incubated in hypotonic 0.75× (9.4%) and 0.5× (15.6%) media compared to 1× media (3.5%), although not to the extent seen in COLO320DM cells (Fig. 3, *D* and *E*). However, there was no significant increase in the percentage of *MYC* FISH signal incorporated into micronuclei in COLO320HSR cells incubated in hypotonic 0.75× (0.6% ± 5.8) and 0.5× (1.0% ± 7.2) media compared to 1× media (0.3% ± 3.9) (Fig. 3*E*), and the micronuclei that were formed mostly did not contain *MYC* FISH signal (Fig. S3*B*). This indicates that hypotonic conditions may affect the segregation of chromosomes or non-ecDNA acentric fragments. These results suggest that untethered ecDNAs are more prone to mis-segregation into micronuclei.

Next, I asked whether HDAC inhibition induces ecDNA micronuclei formation in newly divided daughter cells. TSA treatment was highly cytostatic and prevented cells from completing mitosis; however, at low concentrations, LMK235 and Belinostat treatment allowed cells to progress through mitosis. I treated COLO320DM cells with low concentrations of LMK235 (≤0.3 μM) and Belinostat (≤0.5 μM), which increased histone H3 acetylation in both mitotic chromosomes and ecDNA (Figs. 3*F* and S3*C*). Both inhibitors increased micronuclei formation (41% of daughter cell pairs treated with 0.3 μM LMK235 contained micronuclei compared to 14.7% with vehicle; 58.7% with 0.5 μM Belinostat treatment compared to 11.8% with vehicle) and the percentage of ecDNA incorporated into micronuclei (2.6% with 0.3 μM LMK235 treatment compared to 0.6% with vehicle; 3.0% with 0.5 μM Belinostat treatment compared to 0.7% with vehicle) (Figs. 3, *G* and *H* and S3, *G* and *H*). The micronuclei formed mainly contained ecDNA (Fig. S3, *D* and *I*). On the contrary, COLO320HSR cells treated with the same concentrations of LMK235 showed histone hyperacetylation (Figs. 3*I* and S3*E*) but did not exhibit increased micronuclei formation (Figs. 3, *J* and *K* and S3*F*). These results suggest that ecDNA untethering by histone hyperacetylation promotes ecDNA mis-segregation into micronuclei, similar to the effects observed under hypotonic conditions. This further supports a link between chromatin compaction and ecDNA tethering and segregation.

### Ki67 regulates ecDNA tethering to chromosomes

**Figure 4:**
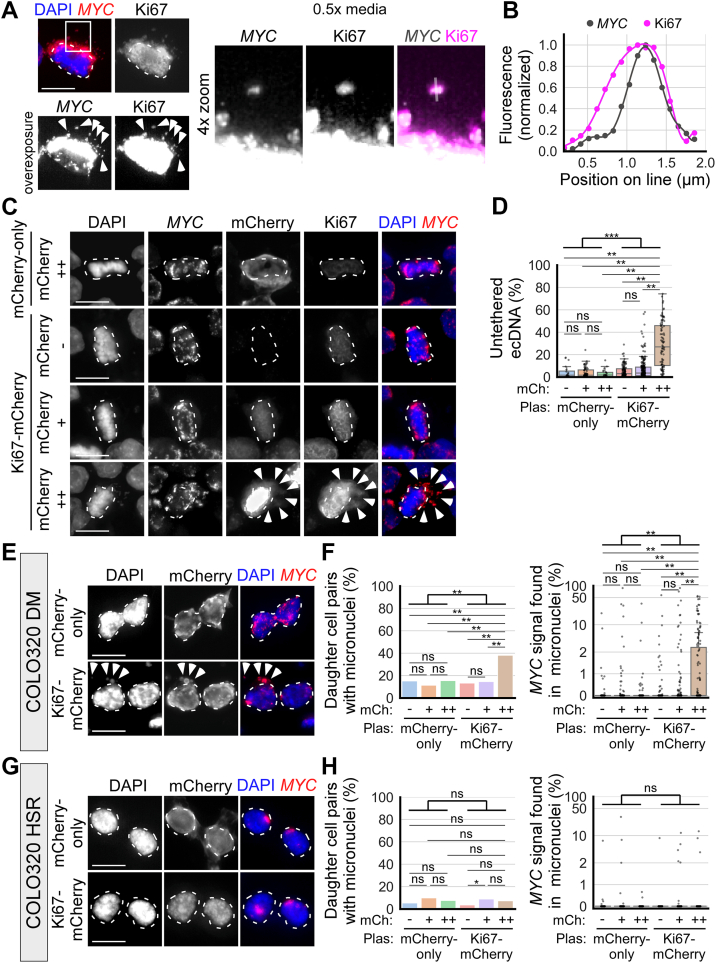
**Ki67 gain-of-function untethers ecDNA from chromosomes.***A*, fixed COLO320DM cells cultured on coverslip and incubated in 0.5× media for 15 min to visualize and colocalize individual ecDNA (*MYC* FISH) with Ki67 IF (*arrowheads*). *B*, line profile of *MYC* FISH and Ki67 IF intensity along the line indicated in *panel* A; polynomial curves were fitted to the line profiles. *C*, metaphase COLO320DM cells cultured on glass coverslips 2 to 4 days post mCherry-only or Ki67-mCherry expression plasmid transfection; cells were categorized based on mCherry fluorescence: - indicates lack of mCherry expression, ++ indicates top 20% tile mCherry expression by fluorescence intensity, + indicates the remaining cells that express mCherry; dashed outlines indicate chromosomes aligned at the metaphase plate, arrowheads indicate untethered ecDNA. *D*, boxplots quantifying ecDNA untethering in plasmid transfected cells from *panel C*; mCh = mCherry expression category, Plas = plasmid transfected; from *left* to *right*; n = 4, 4, 4, 4, 4, 4 biological replicates and 20, 63, 22, 116, 221, 86 total cells; two-way ANOVA, mCherry expression category (- *versus* + *versus* ++): F = 123.9, *p* < 0.001; plasmid transfected (mCherry-only *versus* Ki67-mCherry): F = 40.5, *p* < 0.001; interaction: F = 33.8, *p* < 0.001; ∗∗*p* < 0.01 by Tukey’s HSD, ns = not significant. *E*, newly-divided daughter COLO320DM cells, as identified by the presence of Aurora B staining (not shown), 2 to 4 days post mCherry-only or Ki67-mCherry expression plasmid transfection (mCherry ++ cells are shown); dashed outlines indicate primary nuclei, arrowheads indicate micronuclei. *F*, quantification of *panel E*. *Left*: quantification of the percentage of daughter cell pairs with micronuclei; chi-squared, ∗∗*p* < 0.01. Right: quantification of the percentage of all *MYC* FISH signal per daughter cell pair inside micronuclei (symlog scale, linear ≤2, log >2); two-way ANOVA, mCherry expression category (- *versus* + *versus* ++): F = 7.6, *p* < 0.001; plasmid transfected (mCherry-only *versus* Ki67-mCherry): F = 7.6, *p* = 0.0058; interaction: F = 4.9, *p* = 0.0073; ∗∗*p* < 0.01; n = 7, 7, 7, 7, 7, 7 biological replicates and 93, 312, 104, 179, 400, 147 daughter cell pairs. *G* and *H*, newly-divided daughter COLO320HSR cells, transfected similarly as in *panels E* and *F* (mCherry ++ cells are shown). *H*, *left*: chi-squared, ∗*p* < 0.05. *Right*: two-way ANOVA, mCherry expression category (- *versus* + *versus* ++): F = 0.7, *p* = 0.49; plasmid transfected (mCherry-only *versus* Ki67-mCherry): F = 0.1, *p* = 0.77; interaction: F = 0.9, *p* = 0.39; n = 4, 4, 4, 4, 4, 4 biological replicates and 168, 245, 107, 202, 227, 111 daughter cell pairs. In all panels, scale bar = 10 μm. Except in *panel B*, each data point in graphs represents one cell or daughter cell pair.

Ki67 is a large nuclear protein that localizes to the surface of chromosomes during mitosis, forming a surfactant-like molecular layer that prevents chromosomes from tightly clustering together (49). I hypothesized that Ki67 may modulate ecDNA tethering similarly to its role in chromosome clustering. First, I assessed the association of Ki67 with mitotic ecDNA by colocalizing Ki67 IF signal with *MYC* FISH signal in COLO320DM cells at metaphase (Fig. 4*A*). To facilitate the colocalization analysis, cells were incubated for 15 min in hypotonic 0.5× media to untether ecDNA. *MYC* FISH signal co-localized with Ki67 IF and a line profile across ecDNA shows that Ki67 signal surrounds *MYC* FISH signal (Fig. 4*B*), similar to what was shown for Ki67 and chromosomes in prior studies (48, 49, 50).

Overexpression of Ki67 has been shown to increase the height of the brush-like structure formed by Ki67 at the surface of mitotic chromosomes, further dispersing chromosomes from each other (49). To test whether Ki67 overexpression may untether ecDNA, I transfected plasmid constructs expressing mCherry-tagged Ki67 (Ki67-mCherry) into COLO320DM cells. At 2 to 4 days post transfection, I imaged metaphase cells cultured and fixed on glass coverslips and categorized them based on the level of mCherry expression: mCherry−, indicating no detectable expression; mCherry ++, representing the top 20% in fluorescence intensity; and mCherry +, indicating the remaining mCherry-expressing cells. ecDNA untethering at metaphase was significantly increased in mCherry ++ cells overexpressing Ki67-mCherry (29.0% ± 20.3 untethered, mean ± standard deviation) compared to mCherry ++ cells overexpressing mCherry without Ki67 (mCherry-only; 3.0% ± 3.7) and all other conditions (Fig. 4, *C* and *D*). The percentage of daughter cell pairs containing micronuclei was also increased in cells expressing high levels of Ki67-mCherry (44.9%), compared to mCherry-only (15.4%) (Fig. 4, *E* and *F*). The percentage of ecDNA incorporated into micronuclei was similarly increased (4.6% ± 10.8 in the Ki67-mCherry condition compared to 0.5% ± 3.6 in the mCherry-only condition) in newly divided daughter cells (Fig. 4*F*). The micronuclei formed in cells expressing high levels of Ki67-mCherry mainly contained ecDNA (Fig. S4*A*). On the contrary, no increase in micronuclei formation was observed in COLO320HSR cells expressing high levels of Ki67-mCherry (Figs. 4, *G* and *H* and S4*B*). These results show that Ki67 overexpression untethers ecDNA from chromosomes and promotes their mis-segregation into micronuclei.

As a control for protein overexpression, which may affect intracellular tonicity, I transfected COLO320DM cells with a plasmid construct expressing mCherry-tagged human albumin (mCherry-Albumin), from which the signal peptide sequence is removed, keeping the protein intracellular. Metaphase cells expressing high levels of mCherry-Albumin did not show increased ecDNA untethering (Fig. S4, *C* and *D*) nor increased micronuclei formation (Fig. S4*E*).

Prior studies have shown that the surfactant function of Ki67 and its ability to disperse mitotic chromosomes do not reside within a single domain of the protein, but rather are due to the size and charge of the protein, which, as it coats the surfaces of chromosomes, produces a combination of electrostatic repulsion and steric hindrance preventing inter-chromosomal interactions (48, 49). Ki67 is amphiphilic. The Ki67 C-terminal LR (leucine-arginine rich) domain is highly positively charged and preferentially associates with the surface of mitotic chromatin. The remainder of the protein, including the N-terminal forkhead-associated (FHA) domain, the protein phosphatase 1–binding domain (PP1-BD), and the central region containing 16 repeat domains, associates with the cytosol and prevents tight clustering of chromosomes due to its overall positive charge and large, extended structure (48, 49). To determine if the ability of Ki67 overexpression to untether ecDNA is similarly dependent on the size of the non-LR domain regions of Ki67 rather than a specific domain, I ectopically expressed in COLO320DM cells one of five truncated versions of Ki67 tagged with mNeonGreen (mNG) as previously described (49), to determine whether they are able to untether ecDNA. These truncated versions are: (i) Ki67-ΔFHA, which lacks the FHA domain, (ii) Ki67-ΔNterm, which lacks the FHA and PP1-BD domains, (iii) Ki67-ΔRepeats, which lacks the 16 repeat domains, (iv) Ki67-LR+8Repeats, which lacks the FHA, PP1-BD and 8 repeat domains, and (v) Ki67-LR-only, which lacks all domains except the LR domain (Fig. S4*F*). As a control for protein overexpression, I also expressed the Ki67-LR-only construct fused to intracellular albumin (Albumin-Ki67-LR). Similar to prior findings with chromosome dispersal (49), the expression of Ki67-ΔFHA, Ki67-ΔNterm, and Ki67-ΔRepeats mutants led to ecDNA untethering in COLO320DM metaphase cells and promoted micronuclei formation (Fig. S4, *G*–*I*). The expression of smaller Ki67 truncated mutants (Ki67-LR+8Repeats and Ki67-LR-only) and the Albumin-Ki67-LR construct did not increase ecDNA untethering or micronuclei formation (Fig. S4, *G*–*I*). These results suggest that Ki67 promotes chromosomal dispersal and ecDNA untethering in similar ways that are dependent on its size and charge more than on specific functional domains.

**Figure 5:**
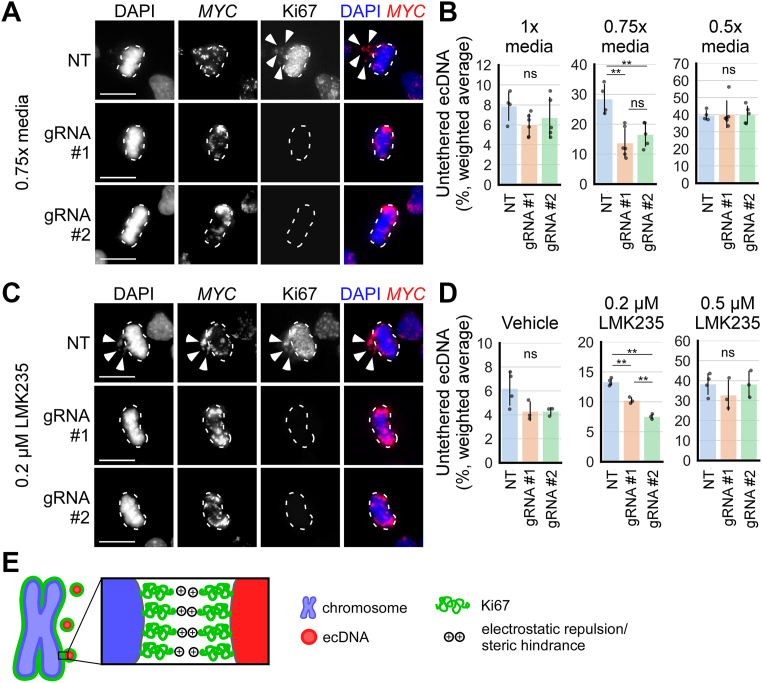
**Ki67 loss-of-function decreases ecDNA untethering from chromosomes.***A*, metaphase COLO320DM wildtype (NT) and *MKI67* knockout cells (gRNA #1 and #2), incubated for 15 min in 1× media (not shown), 0.75× media, and 0.5× media (not shown); dashed outlines indicate chromosomes aligned at the metaphase plate, arrowheads indicate untethered ecDNA. *B*, quantification of ecDNA untethering of COLO320DM wildtype and *MKI67* knockout clones from *panel A* (each data point represents the weighted average of at least 95 cells from each clone) after 15 min incubation in 1× media (*left*; n = 4, 6, 5 clones; one-way ANOVA, F = 1.7, *p* = 0.23), 0.75× media (*middle*; n = 4, 6, 5 clones; one-way ANOVA, F = 12.4, *p* = 0.001), and 0.5× media (*right*; n = 4, 6, 5 clones; one-way ANOVA, F = 0.1, *p* = 0.99); ∗∗*p* < 0.01 by Tukey’s HSD, ns = not significant; error bars = mean ± standard deviation. *C*, metaphase COLO320DM wildtype (NT) and *MKI67* knockout cells (gRNA #1 and #2), treated for 24 h with vehicle (not shown), 0.2 μM LMK235, and 0.5 μM LMK235 (not shown); dashed outlines indicate chromosomes aligned at the metaphase plate, arrowheads indicate untethered ecDNA. *D*, quantification of ecDNA untethering of COLO320DM wildtype and *MKI67* knockout clones from *panel C* (each data point represents the weighted average of at least 60 cells from each clone) after 24 h treatment with vehicle (0.1% DMSO, *left*; n = 4, 3, 3 clones; one-way ANOVA, F = 3.9, *p* = 0.073), 0.2 μM LMK235 (*middle*; n = 4, 3, 3 clones; one-way ANOVA, F = 100.8, *p* < 0.001, ∗∗*p* < 0.01 by Tukey’s HSD), and 0.5 μM LMK235 (*right*; n = 4, 3, 3 clones; one-way ANOVA, F = 0.8, *p* = 0.51); error bars = mean ± standard deviation; *MKI67* knockout clones are gRNA #1 clones 1, 4, and 5, and gRNA #2 clones 1, 4, and 5 (see Fig. S5, *A*–*C*). *E*, schematic: the biological surfactant Ki67 (*green*) coats the surface of mitotic ecDNA (*red*) and chromosomes (*blue*), helping to prevent tethering by electrostatic repulsion and steric hindrance. In all panels, scale bar = 10 μm.

I next investigated the consequences of Ki67 loss-of-function. I deleted *MKI67* (encoding Ki67) in COLO320DM cells using CRISPR-Cas9 gene editing with one of two guide RNAs (gRNA #1 and gRNA #2) and isolated independent clones (Fig. S5, *A* and *B*). Both gRNAs effectively decreased Ki67 protein levels as assessed by western blotting and IF, compared to control cell clones transfected with a non-targeting guide (NT) (Fig. S5, *C* and *D*). Loss of Ki67 slightly but not statistically significantly increased ecDNA tethering (decreased ecDNA untethering) in metaphase COLO320DM cells under isotonic (1× media) conditions (6.0% ± 1.1 untethered with gRNA #1 and 6.7% ± 2.1 with gRNA #2, compared to 7.9% ± 1.5 with NT) (Fig. 5, *A* and *B*). Loss of Ki67 significantly decreased eDNA untethering in metaphase cells incubated for 15 min in hypotonic 0.75× media (13.7% ± 4.9 untethered with gRNA #1 and 16.5% ± 4.1 with gRNA #2, compared to 28.4% ± 5.0 with NT). Interestingly, the effect was not observed in cells incubated in hypotonic 0.5× media (40.3% ± 8.1 untethered with gRNA #1 and 40.1% ± 5.2 with gRNA #2, compared to 39.6% ± 3.0 with NT), suggesting that stronger hypotonic conditions can compensate for the loss of Ki67, promoting a similar amount of ecDNA untethering in Ki67-deleted cells as in control cells (Fig. 5*B*). Similar results were observed in COLO320DM cells in which *MKI67* expression was knocked down with one of two small interfering RNAs (siRNAs; siMKI67 #1 and #2) compared to a non-targeting control (siControl) (Fig. S5, *E*–*G*). In addition, treatment of Ki67-deleted clones with 0.2 μM of the HDAC inhibitor LMK235 for 24 h did not lead to as much ecDNA untethering as wildtype clones (10.2% ± 0.5 untethered with gRNA #1 and 7.5% ± 0.4 with gRNA #2, compared to 13.3% ± 0.6 with NT) (Fig. 5, *C* and *D*). As with hypotonic conditions, treatment with a higher dose of LMK235 (0.5 μM) compensated for the loss of Ki67 (32.7% ± 7.8 untethered with gRNA #1 and 38.2% ± 6.7 with gRNA #2, compared to 38.3% ± 5.4 with NT), promoting ecDNA untethering equally in control and Ki67-deleted cells (Fig. 5*D*). Altogether, these results indicate that the chromosome surfactant Ki67 coats ecDNA and regulates ecDNA tethering to chromosome during mitosis (Fig. 5*E*).

### HDAC inhibition leads to a loss of oncogene copy number in COLO320DM cells, but not in COLO320HSR cells

**Figure 6:**
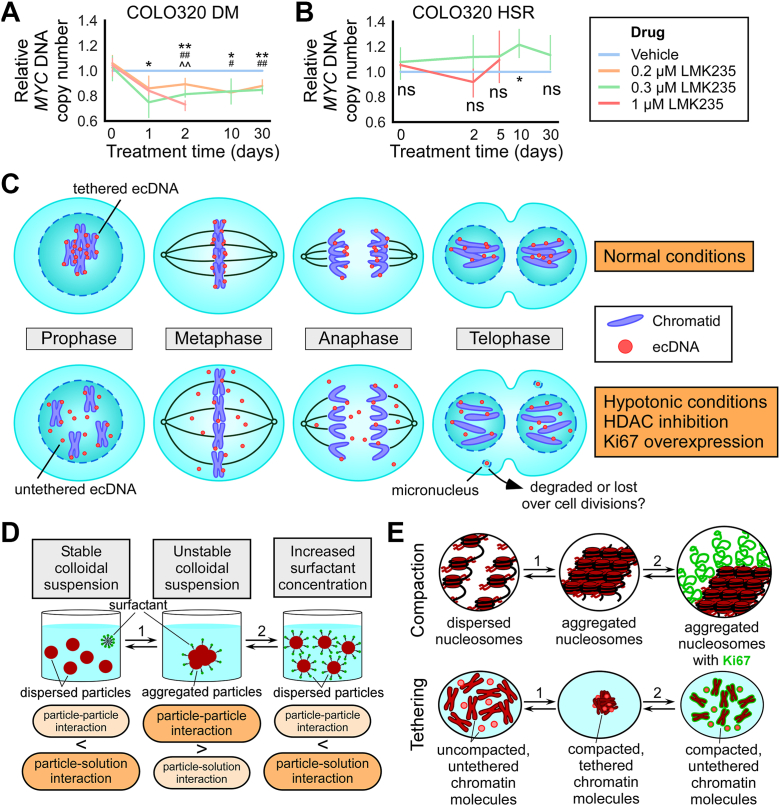
**HDAC inhibition leads to a loss of oncogene copy number in COLO320DM cells, but not in COLO320HSR cells.***A* and *B*, quantification of *MYC* DNA expression in COLO320DM (*A*) and COLO320HSR (*B*) cells treated with LMK235; qPCR, 2^-ΔΔCT^ analysis (normalized to *LINE1* copy number and vehicle control); x-axis = symlog scale, linear ≤2, log >2. Error bars = mean ± 95% confidence interval. Drug-containing media was replaced every 2 to 3 days. *A*, treatment with vehicle (0.1% DMSO), 0.2 μM, 0.3 μM, or 1 μM LMK235 for 1 day (one-way ANOVA, F = 4.1, *p* = 0.036, n = 4, 4, 4, 3), 2 days (F = 31.3, *p* < 0.001, n = 4, 4, 4, 3), 10 days (F = 5.4, *p* = 0.017, n = 6, 6, 6, no 1 μM), 30 days (F = 19.8, *p* = 0.0023, n = 3, 3, 3, no 1 μM); 0 days: pretreatment (one-way ANOVA, F = 0.4, *p* = 0.77, n = 3, 3, 3, 3); #*p* < 0.05, ##*p* < 0.01 (0.2 μM LMK235 *versus* vehicle); ∗*p* < 0.05, ∗∗*p* < 0.01 (0.3 μM LMK235 *versus* vehicle); ˆˆ*p* < 0.01 (1 μM LMK235 *versus* vehicle) by Tukey’s HSD; *B*, treatment with vehicle, 0.3 μM LMK235, or 1 μM LMK235 for 2 days (one-way ANOVA, F = 2.2, *p* = 0.17, n = 4, 4, 4), 5 days (F = 0.5, *p* = 0.62, n = 4, 4, 4), 10 days (F = 10.1, *p* = 0.019, n = 4, 4), and 30 days (F = 2.7, *p* = 0.15, n = 4, 4); 0 days: pretreatment (one-way ANOVA, F = 0.8, *p* = 0.48); ∗*p* < 0.05 (0.3 μM LMK235 *versus* vehicle). *C*, schematic, *top*: under normal mitotic conditions, ecDNA tether to chromosomes throughout mitosis to ensure their segregation into the primary nuclei of divided daughter cells; *bottom*: under conditions that decompact chromatin (hypotonic conditions and HDAC inhibition) or prevent ecDNA-chromosome interaction at their surfaces (Ki67 overexpression), ecDNA untether from chromosomes, leading some to be mis-segregated into micronuclei. *D* and *E*, schematics of a colloidal and surface chemistry-based framework for approaching ecDNA and chromosome compaction and tethering during mitosis. *Arrow* 1 represents biophysical changes to the colloidal particles (nucleosomes and chromatin molecules) or the solution/suspension medium (cytosol), such as alterations to the intracellular ionic strength or to the acetylation state of chromatin. *Arrow* 2 represents changes in the surfactant (Ki67) concentration of the system. Both sets of changes affect particle-particle and particle-solution interactions.

Next, I asked whether ecDNA untethering and incorporation into micronuclei may affect the inheritance of ecDNA over the course of several cell divisions, leading to a decrease in ecDNA copy number over time. I treated COLO320DM cells, which have a doubling time of roughly 24 h (77), with 1 uM of the HDAC inhibitor LMK235 and quantified ecDNA copy number *via MYC* DNA qPCR after 1 and 2 days of treatment. I observed a 27% reduction in *MYC* copy number at day 2, before cells stopped dividing and/or died. A similar reduction in *MYC* copy number (10–20%) was observed at lower concentrations of LMK235 (0.2 and 0.3 μM) up to 30 days of treatment (Fig. 6*A*). No loss of *MYC* copy number was observed in COLO320HSR cells treated with LMK235 (Fig. 6*B*). Rather, there appeared to be a 10 to 20% increase in *MYC* copy number on Day 10 of treatment. Overall, these data suggest that HDAC inhibition decreases ecDNA copy number.

## Discussion

The mechanisms that tether ecDNA to chromosomes during mitosis and safeguard them from mis-segregation into micronuclei are not completely known (22). Mitotic chromosomes are formed in part by global chromatin compaction driven by increased free intracellular Mg^2+^ concentration and histone deacetylation that modulate chromatin-chromatin interactions (25). Consequently, mitotic chromatin can be decondensed by hypotonic conditions and HDAC inhibition (43, 44, 45, 46, 47). Here, I show that ecDNA-chromosome tethering is impaired by hypotonic conditions and histone hyperacetylation, suggesting that tethering is coupled to global chromatin compaction during mitotic chromosome formation. I propose that ecDNA and chromosomes tether to each other *via* chromatin fiber interactions at the surface of the molecules, mediated by electrostatic and/or hydrophobic forces that also drive mitotic chromatin compaction.

The surfactant Ki67 coats the surface of mitotic chromosomes to help disperse them during mitosis (48, 49, 50, 51). I show that Ki67 also acts similarly to untether ecDNA from chromosomes. Furthermore, Ki67 depletion negates the ecDNA untethering effects of conditions that decompact mitotic chromatin, *i.e.*, incubation in hypotonic 0.75× media and HDAC inhibition *via* low dose (0.2 μM) LMK235 (Fig. 5, *A*–*D*). Interestingly, I found an “epistatic” interaction between hypotonic conditions, histone hyperacetylation by HDAC inhibition, and Ki67, as a strong hypotonic condition (0.5× media) and high concentrations of LMK235 (0.5 μM) can compensate for the lack of Ki67, producing high levels of ecDNA untethering despite the lack of Ki67. This “epistatic” interaction shows that global chromatin compaction and Ki67-mediated chromatin surface regulation, two of the mechanisms known to help form and organize mitotic chromatin (25), act together to coordinate ecDNA tethering. I propose that Ki67 disrupts ecDNA-chromosome tethering by preventing chromatin-chromatin interactions at the surface of the molecules that would otherwise take place because of mitotic chromatin compaction.

The finding that ecDNA tethering is coupled with mitotic chromatin compaction suggests that tethering-specific protein complexes and mechanisms are not necessary for the nuclear inheritance of all acentric chromatin. However, the present study does not rule out a role for chromatin-associated protein complexes in ecDNA tethering. Proteins and protein complexes are involved in tethering various forms of acentric chromatin to mitotic chromosomes, including viral DNA, contributing to their persistence (75, 78). Recent studies have shown that RNA polymerase and bromodomain proteins such as BRD4 have a role in ecDNA tethering in cancer cells (23, 24). It is possible that hypotonic conditions used in the present study disrupt the association of these proteins with chromatin, which could be an alternative explanation for the observed ecDNA untethering. However, histone hyperacetylation from HDAC inhibition would be predicted to increase BRD4 binding to chromatin to potentially increase tethering rather than cause untethering as observed in the present study. Other protein complexes that may be involved in ecDNA tethering include molecular motors that organize mitotic chromosomes *via* DNA loop extrusion, such as condensin (26). The potential role of these molecular motors in organizing mitotic ecDNA and tether ecDNA to chromosomes will be investigated in future studies. I propose that mitotic chromatin compaction provides a baseline level of ecDNA tethering that protein-complex-mediated tethering may act on top of to further stabilize mitotic ecDNA-chromatin associations. Therefore, I propose that the same compaction-mediated tethering mechanism plays a role in the segregation of other forms of acentric chromatin, including acentric chromosomal fragments and chromatinized viral DNA, which will be topics of future studies. I hypothesize that the size of the acentric molecules may be a factor in determining whether tethering to chromosomes ensures proper nuclear segregation, as larger molecules experience more drag during mitotic movements in the cytosol, which may act in an opposing direction to tethering forces. This may explain why chromatin molecules much larger than a single ecDNA, such as lagging chromosomes and large aggregates of ecDNA formed due to DSB, often untether and mis-segregate into micronuclei under normal mitotic conditions (59, 60, 79).

Each of the conditions that untether ecDNA at metaphase, *i.e.*, hypotonic conditions, HDAC inhibition, and Ki67 overexpression, also increase mis-segregation of ecDNA into micronuclei (Fig. 6*C*). While this suggests that untethered ecDNA are more likely to be mis-segregated, alternative mechanisms of ecDNA incorporation into micronuclei cannot be ruled out. Additionally, DNA inside micronuclei have been suggested to be degraded or lost over several cell divisions (80, 81, 82). The finding that HDAC inhibition reduces ecDNA copy number over time suggests that ecDNA within micronuclei may be lost over cell divisions due to replication/repair defects or degradation (83). However, more studies are required to determine the exact mechanism underlying the copy number loss.

Both HDAC inhibition (84) and modulation of the extracellular tumor environment *via* hypotonic solutions (85) have been used or are potential methods for cancer treatment. I show here that while these treatments may help rid cells of ecDNA, they also cause ecDNA mis-segregation into micronuclei. As mis-segregation and micronuclei formation have been shown to be significant contributors to cancer genomic heterogeneity, progression, and metastasis (83), the costs and benefits of treatments involving HDAC inhibition and hypotonic solutions on ecDNA-containing cancers should be carefully considered and further studied.

Finally, I propose a framework for approaching mitotic chromosome and ecDNA tethering based on colloidal and surface chemistry (Fig. 6*D*). In a stable colloidal suspension of solid particles in a liquid medium, such as a liquid solution, particles are dispersed within the solution, as the particle-particle interaction is weaker than the particle-solution interaction (86). However, upon changes to the biophysical properties of the particles or the solution (arrow 1), the colloidal suspension may become unstable, where particle-particle interactions are stronger than particle-solution interactions. This causes the particles to aggregate, maximizing particle-particle interactions while minimizing particle-solution interactions. Amphiphilic surfactants that stabilize the particle-solution interface help prevent particle aggregation. An increase in surfactant concentration (arrow 2) can disperse the aggregated particles (86). Chromatin suspended in the mitotic cytosol can be modeled as a colloidal suspension at two levels: 1) the level of nucleosomes and 2) the level of chromatin molecules, *i.e.* chromosomes and ecDNA (Fig. 6*E*). Nucleosomes of decompacted chromatin are dispersed in the cytosol (Fig. 6*E*, top). However, upon an increase in the cytosolic salt/ion concentration or histone deacetylation by HDACs (arrow 1), nucleosomes aggregate, leading to chromatin compaction. This process can be reversed by hypotonic conditions and HDAC inhibition. Zooming out (Fig. 6*E*, bottom), decompacted mitotic chromosomes and ecDNA are dispersed in cytosol, but upon changes that lead to chromatin compaction (arrow 1), they tether to each other due to the same biophysical forces responsible for nucleosome aggregation occurring at the surface of the molecules. This process can be similarly reversed to untether the chromatin molecules. The surfactant Ki67 is large (∼90 nm in length) and acts at the surface of chromatin molecules to stabilize the chromatin-cytosol interface (49). An increase in the concentration of Ki67 (arrow 2) can untether the chromatin molecules. This framework argues that a complex biological phenomenon such as ecDNA tethering and mitotic inheritance can at least be partially explained by a relatively simple biophysical model of the molecules and their surrounding milieu.

## Experimental procedures

### Cell lines and cell culture

Human colorectal cancer cell lines COLO320DM and COLO320HSR were purchased from ATCC. COLO320DM and HSR cell lines were derived from colorectal carcinoma (87). Cell line authenticity was confirmed by short tandem repeat profiling. Cells were cultured in Dulbecco’s Modified Eagle’s Media with 4.5 g/L Glucose, L-glutamine, and Sodium Pyruvate (Corning) supplemented with 10% fetal bovine serum (Gibco A5256701). Cells were maintained at 37 °C in a humidified incubator with 5% CO_2_. Cell lines routinely tested negative for *mycoplasma* contamination. For experiments examining ecDNA untethering at metaphase, cells were treated for 3 h with 10 μM of the proteasome inhibitor MG132 to enrich for the proportion of cells in metaphase (66).

### Chemicals and inhibitors

The following chemicals were used in the study: MG132 (Selleck S2619), Colcemid (Gibco 15,210–040), Trichostatin A (MedChemExpress HY-15144), Belinostat (MedChemExpress HY-10225), Abexinostat (PCI-24781, MedChemExpress HY-10990), Fimepinostat (CUDC-907, MedChemExpress HY-13522), Pracinostat (SB939, MedChemExpress HY-13322), LMK235 (MedChemExpress HY-18998), TMP269 (MedChemExpress HY-18360), Mocetinostat (MGCD0103, MedChemExpress HY-12164), Entinostat (MS275, Tocris 6208), RGFP966 (Cayman 16917), Santacruzamate A (CAY10683, Cayman 15,403), Tasquinimod (MedChemExpress HY-10528), BRD73954 (MedChemExpress HY-18700), Nicotinamide (Cayman 11127), EX527 (Cayman 10009798), CTPB (Cayman 19570), 5-Azacytidine (Sigma Aldrich A2385), GSK343 (Selleck S7164), RRx001 (Selleck S8405), ZM447439 (Selleck S1103), Hesperadin (Selleck S1529), Paprotrain (Selleck E0779), and DAPI (Invitrogen D1306).

### Antibodies

The following antibodies were used in the study: rabbit anti-H3ac, pan-acetyl (ActiveMotif 39,140; RRID:AB_2793714), used at 1:200 for IF; rabbit anti-phospho-H2A.X, Ser139 (Cell Signaling 9718; RRID:AB_2118009), used at 1:250 for IF; rabbit anti-Aurora B kinase (Bethyl A300–431A; RRID:AB_420938), used at 1:200 for IF; mouse anti-Aurora B kinase (Novus NBP2-50039; RRID:AB_2895237), used at 1:2000 for IF; mouse anti-Ki67 (eBioscience 14-5699-82; RRID:AB_2016711), used at 1:500 for IF, 1:1000 for WB; rabbit anti-mCherry (abcam 167453; RRID:AB_2571870), used at 1:500 for IF; mouse anti-mNeonGreen (Proteintech 32f6; RRID:AB_2827566), used at 1:500 for IF; mouse anti-Actin (Sigma A4700; RRID:AB_476730), used at 1:10,000 for WB; anti-mouse IgG and anti-rabbit secondary antibodies, Alexa Fluor (Invitrogen), used at 1:500 for IF; anti-mouse IgG, HRP-linked (Cell Signaling 7076S; RRID:AB_330924), used at 1:10,000 for WB.

### Plasmids

Plasmids were transfected into cells using Lipofectamine 3000 Transfection Reagent (Thermo L3000001) following manufacturer’s instructions.

Ki67-ΔFHA expression: Ki67ΔFHA-mNeonGreen-IRESpuro2 was a gift from Daniel Gerlich (Addgene plasmid # 183739; http://n2t.net/addgene:183739; RRID:Addgene_183739). Ki67-ΔNterm expression: Ki67ΔNterm-mNeonGreen-IRESpuro2 was a gift from Daniel Gerlich (Addgene plasmid # 183740; http://n2t.net/addgene:183740; RRID:Addgene_183740). Ki67-ΔRepeats expression: Ki67Δrepeats-mNeonGreen-IRESpuro2 was a gift from Daniel Gerlich (Addgene plasmid # 183742; http://n2t.net/addgene:183742; RRID:Addgene_183742). Ki67-LR+8Repeats expression: Ki67 (8 repeats + LR)-mNeonGreen-IRESpuro2 was a gift from Daniel Gerlich (Addgene plasmid # 183741; http://n2t.net/addgene:183741; RRID:Addgene_183741). Ki67-LR-only expression: Ki67 (LR)-mNeonGreen-IRESpuro2 was a gift from Daniel Gerlich (Addgene plasmid # 183743; http://n2t.net/addgene:183743; RRID:Addgene_183743).

New plasmid constructs were generated using Gibson assembly. Briefly, assembly fragments were generated either by restriction digest or PCR amplification with Q5 high-fidelity DNA polymerase (New England Biolabs M0491), following the manufacturer’s protocols. Gibson assembly was performed using NEBuilder HiFi DNA Assembly Reaction Master Mix (New England Biolabs E2621) following the manufacturer’s protocols. The assembly reactions were used to transform One Shot Stbl3 Chemically Competent Cells (Invitrogen C7373). For Ki67-mCherry expression, Ki67-mCherry-IRESpuro2 plasmid was generated by replacing mNeonGreen with mCherry coding sequence in Ki67-mNeonGreen-IRESpuro2 (a gift from Daniel Gerlich, Addgene plasmid # 183737; http://n2t.net/addgene:183737; RRID:Addgene_183737). For mCherry-only expression, mCherry-IRESpuro2 plasmid was generated by replacing Ki67-mNeonGreen with mCherry coding sequence in Ki67-mNeonGreen-IRESpuro2 pIRESpuro2-mCherry. For mCherry-Albumin expression, mCherry-albumin-IRESpuro2 plasmid was generated by fusing human serum albumin coding sequence (from pGEM-Alb, SinoBiological HG10968-G) without the signal peptide sequence downstream of mCherry (removing TAA from mCherry) in mCherry-IRESpuro2. For Albumin-Ki67-LR expression, Albumin-Ki67(LR)-mNeonGreen-IRESpuro2 plasmid was generated by fusing human serum albumin coding sequence without the signal peptide sequence in-frame upstream of Ki67-LR in Ki67(LR)-mNeonGreen-IRESpuro2.

### *MKI67* siRNA knockdown

Predesigned Dicer-Substrate siRNAs from TriFECTa RNAi Kit (IDT hs.Ri.MKI67.13) were used, including non-targeting siRNA (siControl), hs.Ri.MKI67.13.1 (siMKI67 #1), and hs.Ri.MKI67.13.2 dsiRNAs (siMKI67 #2), both targeting exon 13 of *MKI67* (NM_002417). Cells were forward transfected using Lipofectamine RNAiMAX Transfection Reagent (Invitrogen 13778075) at an siRNA concentration of 10 nM, following the manufacturer’s instructions. Knockdown efficiency was confirmed by qRT-PCR and IF.

### *MKI67* knockout cell line generation

CRISPR/Cas9 gene editing: predesigned Alt-R CRISPR-Cas9 guide RNAs (gRNA) were used, including Hs.Cas9.MKI67.1.AC (gRNA #1, targeting exon 2 and Hs.Cas9.MKI67.1.AA (gRNA #2), targeting exon 6 *MKI67* (NG_047061.1). Non-targeting gRNA (NT) sequence (GAGAGTGCGCCTTGATAGTA) was selected from a published list (88) based on sequence similarity to the targeting gRNAs. Alt-R S.p. Cas9-RFP V3 enzyme (IDT 10008162) and gRNA ribonucleoprotein (RNP) complex was formed following manufacturer’s instructions and forward transfected using Lipofectamine CRISPRMAX (Invitrogen CMAX00003) at a final RNP concentration of 10 nM, following manufacturer’s instructions. Single cell cloning by limiting dilution: at 48 h post RNP transfection, editing efficiency was confirmed *via* heteroduplex formation and T7 endonuclease I digestion using the Alt-R Genome Editing Detection Kit (IDT 1075931) following manufacturer’s instructions. PCR primers: *MKI67* exon 2 F 5′-GACTTGACGAGCGGTGGTTC-3′, R 5′-GCACCAAGGAAAAGTGACGG-3′; *MKI67* exon 6 F 5′-TGACCACTAGCTCCCAACTG-3′, R 5′-AGGCATCTGTCTGTCGTTGAC-3′. Cells were diluted to 0.5 cell/100 μl in conditioned media and seeded at 100 μl per well in a 96 well plate. Cells were then monitored for single colony formation. Once wells with single colonies reached 50% confluency, cells were transferred to a 24 well plate. gDNA was harvested from each clone, PCR amplified and submitted for Sanger sequencing (Azenta Life Sciences) to confirm mutations and clonal purity. Mutations were quantitated using TIDE (https://tide.nki.nl/) (89). Knockout efficiency was confirmed by IF and Western blotting.

### Quantitative PCR (qPCR and qRT-PCR)

*MYC* copy number enumeration: gDNA was harvested from cells using QIAamp DNA Mini Kit (Qiagen 51304), following manufacturer’s instructions. qPCR was performed using iQ SYBR Green Supermix (Bio-Rad 1708880) following manufacturer’s instructions. *MYC* primers: F 5′-AGCGACTCTGGTAAGCGAAG-3′, R 5′-TGGCCCGTTAAATAAGCTGC-3′. *LINE-1* was used for normalization; primers: F 5′-AAAGCCGCTCAACTACATGG-3′, R 5′-TGCTTTGAATGCGTCCCAGAG-3′. 2^-ΔΔCT^ method was used for analysis.

*MKI67* qRT-PCR: RNA was harvested from cells using RNeasy Mini Kit (Qiagen 74106), following manufacturer’s instructions. cDNA was synthesized using SuperScript VILO cDNA Synthesis Kit (Invitrogen 11754050) following manufacturer’s instructions. qPCR was performed as above. *MKI67* primers: F 5′-CGGTCCCTGAAAATAAGGGAATATC-3′, R 5′-TCTGGGGAGGTCTTCATGGG-3′. *GAPDH* was used for normalization; primer sequences were obtained from a prior publication (90): F 5′-TTGGCTACAGCAACAGGGTG-3′, R 5′-GGGGAGATTCAGTGTGGTGG-3′. 2^-ΔΔCT^ method was used for analysis.

### Western blotting (WB)

Cell lysis: cells were washed with 1× PBS and lysed in 1× RIPA buffer (Thermo 89901) with protease and phosphatase inhibitors (Thermo 1861281) for 30 min on ice with gentle agitation. After centrifugation at 4 °C, 14000*g* for 15 min, the supernatant was flash frozen in liquid nitrogen and stored at −80 C until needed. Protein concentration was determined using the Pierce BCA Protein Assay Kit (Thermo 23225). Gel electrophoresis and transfer: cell lysates mixed with Laemmli sample buffer (Bio-Rad 1610747) and 2-mercaptoethanol (Aldrich M6250) and separated on Protean TGX stain-free gels (Bio-Rad 456-8123). Trans-blot turbo transfer pack (Bio-Rad 1704159) was used to transfer the protein onto a 0.2 μm nitrocellulose membrane. Antibody staining: after 1 h blocking in TBS + 0.1% Tween (TBST) with 3% bovine serum albumin, the membrane was incubated in the primary antibody diluted in blocking solution overnight at 4 °C with gentle agitation. After washing in TBST, the membrane was then incubated in HRP-conjugated secondary antibody diluted in TBST, washed in TBST, and then visualized using the SuperWest Pico PLUS Chemiluminescent kit (Thermo 34579). Blots were imaged using the ImageQuant 800 imager (Amersham). Inverted dark field images (chemiluminescence) were superimposed with bright field images (protein ladder and membrane) using the ImageQuant 800 software.

### Prometaphase spreads, drop method

For performing FISH on prometaphase spreads (Figs. 1, *B* and *D* and S2*F*), cells were incubated for 3 h in 0.1 μg/ml colcemid for 3 h, then washed with 1× PBS and transferred to Eppendorf tubes. Cells were then resuspended in 75 mM KCl (or the indicated solution) and incubated at 37 °C for 15 min with agitation. Cells were then fixed with Carnoy’s fixative (3:1 mix of methanol: glacial acetic acid) added dropwise, followed by three more exchanges of fresh Carnoy’s fixative. Fixed cells were dropped from 10 cm onto a humidified slide and allowed to dry before staining.

### Prometaphase spreads, cytospin method

For immunostaining of prometaphase spreads (Figs. S1, *B* and *C* and S3, *C* and *E*), cells were incubated for 3 h in 0.1 μg/ml colcemid (Gibco 15210-040) for 3 h, then washed with 1× PBS and transferred to Eppendorf tubes. Cells were then gently resuspended in ice-cold hypotonic buffer (10 mM Tris-Cl (pH 7.5) + 20 mM NaCl + 5 mM KCl + 1 mM CaCl_2_ + 0.5 mM MgCl_2_ + 40 mM glycerol) and incubated on ice for 10 min. ∼10k cells were cytospun in 200 μl of hypotonic buffer + 0.25% Tween-20 for each slide at 800 RPM for 8 min.

### Immunofluorescence staining (IF)

Cells cultured on glass coverslips: cells were cultured on glass coverslips coated with 10 μg/ml human plasma fibronectin (Millipore FC010). After fixation in 4% PFA, cells were permeabilized for 10 min in 1× PBS + 0.25% Triton X-100, then blocked for 1 h in 1× PBS + 3% bovine serum albumin. Cells were then incubated with primary antibody diluted in blocking solution at 4 °C overnight. After washing with 1× PBS + 0.1% Tween-20, cells were incubated in fluorescent-dye conjugated secondary antibody diluted in blocking solution. After washing, cells were counterstained with DAPI before mounting.

Cells on cytospin slides: cells fixed in 4% PFA were permeabilized and blocked with DAKO antibody diluent (Agilent S080983-2) + 5 mM MgCl_2_ + 0.2% Triton X-100 for 10 min. Cells were then incubated with primary antibody diluted in blocking solution at 4 °C overnight. After washing with blocking solution + 0.1% Tween-20, cells were incubated in fluorescent-dye conjugated secondary antibody diluted in blocking solution. After washing, cells were counterstained with DAPI before mounting.

### Fluorescence *in situ* hybridization (FISH)

Slides with specimen were dehydrated in an ethanol series (70%, 85%, 100%) and air-dried. Fluorescently-tagged *MYC* probe (Empire Genomics) diluted in hybridization buffer was applied over the specimen and spread using a coverslip. The slides were then warmed to 75 C for 3 min to denature DNA before incubating in a humidified chamber at 37 °C overnight. Slides were washed in 0.4× SSC, followed by 2× SSC + 0.1% Tween-20, and then counterstained with DAPI before mounting.

Dual IF-FISH: after IF staining, cells were fixed in 4% PFA and then permeabilized with 1× PBS + 0.7% Triton X-100 + 0.1 M HCl for 10 min. Cells were then incubated in 1.9 M HCl for 30 min to denature DNA. Cells were then dehydrated in an ethanol series (70%, 85%, 100%) and air-dried. FISH probe was diluted in hybridization buffer and heated to 75 °C for 3 min, then allowed to cool briefly before being applied over the specimen and spread using a coverslip. Slides are then placed in a humidified chamber at 37 °C overnight. Slides were washed in 0.4× SSC, followed by 2× SSC + 0.1% Tween-20, and then counterstained with DAPI before mounting.

### Microscopy

Fluorescence microscopy was performed on a Leica DMi8 widefield microscope by Las X software (v.3.8.2.27713) using a x63 oil objective. Z stacks were acquired covering the entire thickness of cells, with 0.5 μm step size. For live cell imaging, cells were cultured in FluoroBrite media (Gibco A18967–01) in 96 well glass-bottom plates and imaged on a Leica DMi8 widefield microscope using a x63 oil objective at 37 °C with 5% CO_2_.

### Image analysis

Except where indicated below, all image analysis steps were automated using custom Python scripts and algorithms from the Python libraries NumPy (https://numpy.org/), OpenCV (cv2, https://opencv.org/), and scikit-image (skimage, https://scikit-image.org/). Default parameters were used for algorithms unless otherwise specified. Custom graphical user interface (GUI) software for region of interest selection and manual segmentation mask correction was created using the Python library tkinter. All custom scripts are available on github: image processing (https://github.com/yanglum/extract_images_from_lif_file), analyses of prometaphase spread (https://github.com/yanglum/metaseg_gui), analyses of fixed cells cultured on glass coverslips (https://github.com/yanglum/ecDNA_stain_analysis_scripts). For image segmentation (either using the metaseg tool from ecSeg or Otsu’s thresholding method), segmentation masks were visually validated for accuracy.

#### Post-acquisition image processing

For prometaphase spread images, the best in-focus z plane was selected from z-stacks based on Laplacian variance (using the cv2.Laplacian algorithm) for further image analysis. For live cells and fixed cells cultured on glass coverslips, image deconvolution using Leica’s Lightning and Thunder processing was performed, and max projection images were made from z-stacks.

#### ecDNA untethering and chromosome individualization in prometaphase spreads

Prometaphase spreads were segmented using the metaseg tool from ecSeg (https://github.com/UCRajkumar/ecSeg) (56) to distinguish between ecDNA and chromosomes based on DAPI staining, with visual validation and manual correction of the segmentation mask, as needed (with a custom tool, metaseg_gui, https://github.com/yanglum/metaseg_gui), using *MYC* FISH to identify tethered ecDNA. Connected component (CC) analysis was then performed (using the skimage.measure.label algorithm [connectivity = 2] and skimage.measure.regionprops algorithm) on the segmentation mask to quantify ecDNA and chromosome count per prometaphase cell. The percentage of ecDNA untethering is calculated as the number of untethered ecDNA CCs divided by the total number of ecDNA CCs (untethered ecDNA CCs + tethered ecDNA CCs) using a custom script (https://github.com/yanglum/metaseg_gui/blob/main/prometaphase_spread_ecSeg_Analyze_tethering.py). Briefly, untethered ecDNA is defined as ecDNA CC with <25% of its perimeter adjacent to pixels classified as chromosome. Tethered ecDNA is defined as ecDNA CC with 25% to 99.9% of its perimeter adjacent to chromosome pixels. ecDNA CCs with 100% of its perimeter adjacent to chromosome pixels are not considered in the quantification. The number of chromosome CCs per prometaphase cell is used to represent the amount of chromosome individualization. For the inhibitor screen in Figures 2*F* and S2, *F* and *G*, DAPI and *MYC* FISH signal were segmented by Otsu’s thresholding method (using the skimage.filters.threshold_multiotsu algorithm [classes = 3]; the higher threshold was used for segmentation) and the convex hull area was calculated (using the skimage.morphology.convex_hull_image algorithm) based on the segmentation for each cell (union of DAPI and *MYC* FISH segmentation). The convex hull area is used as an estimate of ecDNA and chromosome untethering.

#### pan-H3ac immunofluorescence quantification in prometaphase spreads

prometaphase spreads were segmented using the metaseg tool from ecSeg as above. Average pan-H3ac immune- and DAPI fluorescence intensity were quantified for all pixels classified as either chromosome or ecDNA per cell using a custom script (https://github.com/yanglum/metaseg_gui/blob/main/prometaphase_spread_ecSeg_Analyzing_IF_stain.py). Pan-H3ac fluorescence is normalized to DAPI fluorescence to control for DNA content (ecDNA have lower DAPI staining intensity than chromosomes).

#### ecDNA untethering and micronuclei quantification in fixed cells cultured on glass coverslips

Analyses were performed using custom scripts (https://github.com/yanglum/ecDNA_stain_analysis_scripts), specifically: cells were segmented for DAPI and *MYC* FISH signal by Otsu’s thresholding method (using the skimage.filters.threshold_multiotsu algorithm [classes = 3]; the higher threshold was used for segmentation of FISH and metaphase DAPI signal; the lower threshold was used for segmentation of interphase DAPI signal). For quantification of ecDNA untethering, metaphase cells were manually identified on DAPI based on the appearance of chromosomes aligned at the metaphase plate. The largest DAPI connected component (CC) was labeled as the genomic mass of chromosomes aligned at the metaphase plate (CC analysis was performed using the skimage.measure.label algorithm [connectivity = 2] and the skimage.measure.regionprops algorithm). *MYC* FISH positive pixels overlapping with the genomic mass were quantitated as tethered, while those not overlapping with the genomic mass were quantitated as untethered. Percentage of untethered ecDNA was calculated as the number of untethered *MYC* FISH positive pixels divided by the total number of *MYC* FISH positive pixels per cell. For micronuclei analysis, newly divided daughter cells were manually identified based on the presence of Aurora B kinase IF between the two cells (*e.g.*, Fig. 3*B*). DAPI segmentation was used as the segmentation mask with the primary nuclei and micronuclei were manually identified and labeled. Micronuclei were defined as smaller areas of DAPI signal found outside of the large primary nuclei. Percentage of *MYC* signal found in micronuclei was calculated as the number of *MYC* FISH positive pixels segmented as micronuclei divided by the total number of *MYC* FISH positive pixels between both daughter cells. Micronuclei composition (*MYC*/DAPI %) was calculated as the number of *MYC* FISH-positive pixels segmented as micronuclei divided by the number of DAPI positive pixels segmented as micronuclei between both daughter cells. For pH2A.X foci quantification, pH2A.X IF signal was segmented by Otsu’s thresholding method (as described previously; the higher threshold was used for segmentation) and the number of foci per cell was quantified using CC analysis (as described above), where only CCs consisting of more than five pixels were counted. For IF signal quantification (Ki67, mCherry, mNeonGreen), the IF signal was segmented by Otsu’s thresholding method (as described previously; the lower threshold was used for segmentation) and the total IF signal (integrated density) per cell was quantified within the segmented region.

### Graphing and statistics

All graphing and statistical analyses were performed in Python using the libraries NumPy, pandas (https://pandas.pydata.org/), matplotlib (https://matplotlib.org/), seaborn (https://seaborn.pydata.org/), statsmodels (https://www.statsmodels.org/: ANOVA [statsmodels.api.anova_lm, typ = 2], Tukey’s HSD [statsmodels.stats.multicomp.pairwise_tukeyhsd]) and SciPy stats (https://scipy.org/: linear regression [scipy.stats.linregress], Student’s *t* test [scipy.stats.ttest_ind], chi-squared [scipy.stats.chi2_contingency]). All statistical methods and sample sizes can be found in the figure legends. No statistical methods were used to predetermine the sample size. For ANOVA, Tukey’s honestly significant difference (HSD) was used for posthoc analysis when *p* < 0.05.

## Data availability

All data supporting the findings of this study are available upon reasonable request (yanglum@stanford.edu).

## Supporting information

This article contains supporting information (49).

## Conflict of interest

The authors declare that they have no conflicts of interest with the contents of this article.
