# Supplementary material for: Mitotic chromatin compaction tethers extrachromosomal DNA to chromosomes and prevents their mis-segregation into micronuclei
Source: J Biol Chem. 2025 Dec 20;302(2):111081. doi: 10.1016/j.jbc.2025.111081 (PMC12857182; doi:10.1016/j.jbc.2025.111081)
Supplement: Supplemental_information [file mmc1.pdf]

Supplemental Information for

Mitotic chromatin compaction tethers extrachromosomal DNA to chromosomes and prevents their mis-segregation into micronuclei

Lu M. Yang

Materials included:

Supplemental Figures S1-S5

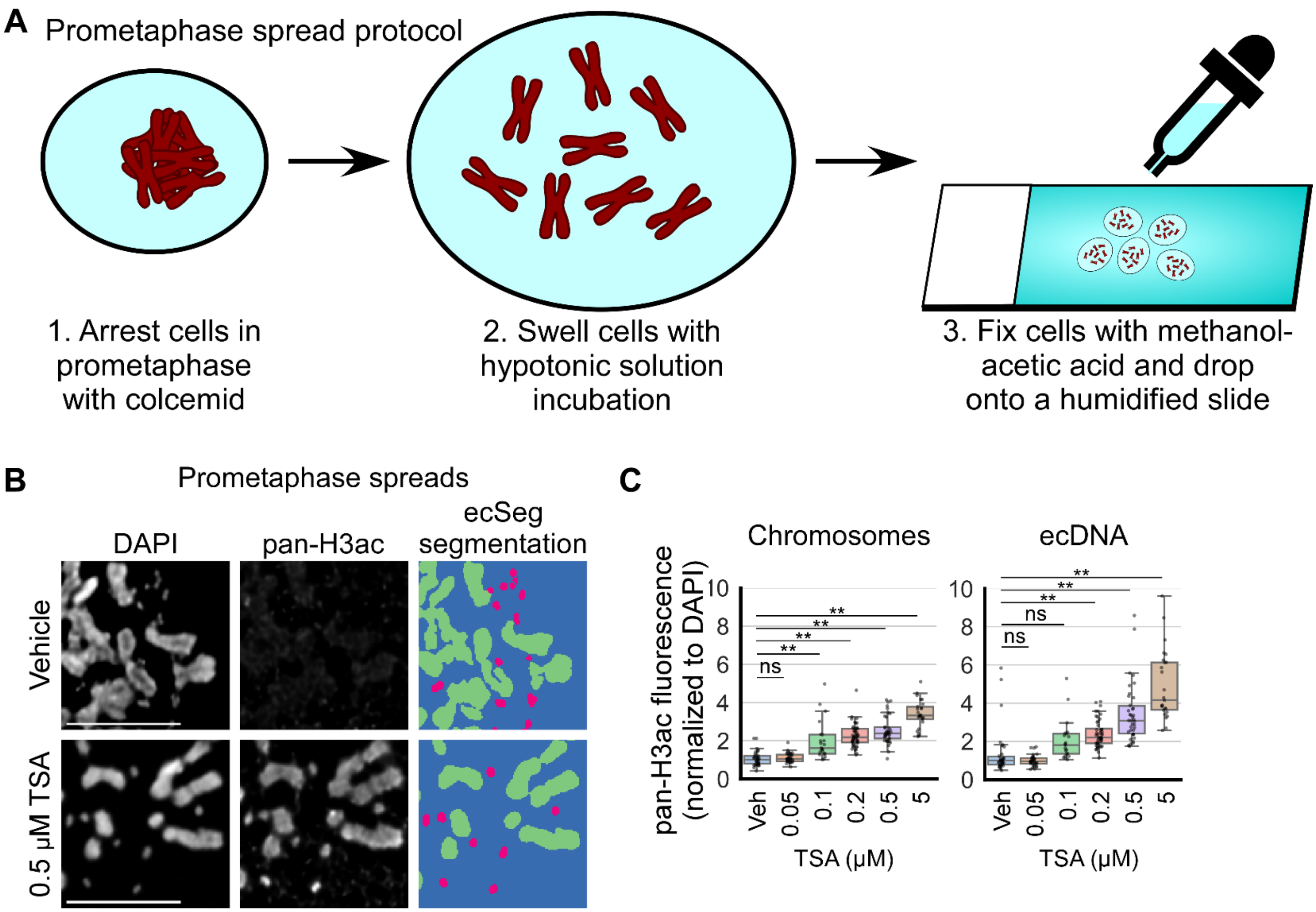

Figure S1. Related to Fig. 1.

A, the prometaphase spread technique involves three main steps. 1) Cells are arrested in prometaphase by treatment with a mitotic spindle destabilizing drug, such as colcemid. 2) Cells are incubated in hypotonic solution, conventionally 75 mM KCl. 3) Cells are fixed with a 3:1 mixture of methanol and acetic acid and dropped onto a humidified slide. ***B,*** cytospin preparations of prometaphase-arrested COLO320DM cells incubated in hypotonic solution, with IF staining for pan-Histone H3 acetylation (pan-H3ac). ecSeg was used to classify pixels as either chromosome (green) or ecDNA (red). Scale bar = 10 µm. ***C,*** boxplots quantifying pan-H3ac IF signal by integrated density from panel B, normalized to DAPI signal, for chromosomes (left) and ecDNA (right); from left to right, n=3, 1, 1, 1, 2, 2, biological replicates and 34, 33, 21, 48, 38, 25 cells. Left: one-way ANOVA, F=60.8, p<.001. Right: one-way ANOVA, F=44.1, p<.001; **p<.01 by Tukey's HSD, ns = not significant. Each data point represents one cell.

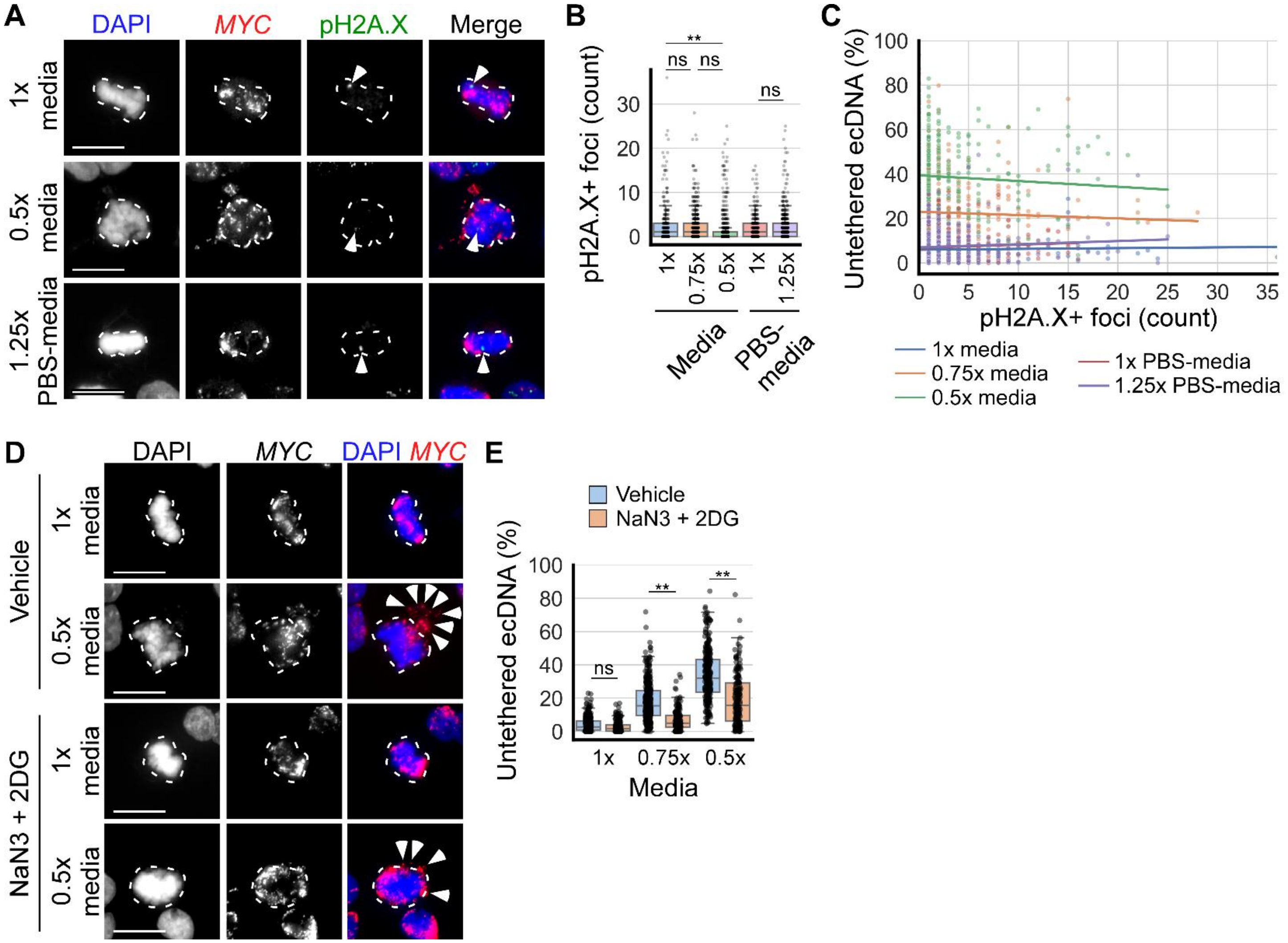
A
DAPI
MYC
pH2A.X
Merge
1x media
0.5x media
1.25x PBS-media
B
pH2A.X+ foci (count)
**
ns
ns
ns
1x
0.75x
0.5x
1x
1.25x
Media
PBS-media
C
Untethered ecDNA (%)
pH2A.X+ foci (count)
1x media
0.75x media
0.5x media
1x PBS-media
1.25x PBS-media
D
DAPI
MYC
DAPI MYC
Vehicle
NaN3 + 2DG
1x media
0.5x media
E
Vehicle
NaN3 + 2DG
Untethered ecDNA (%)
ns
**
**
1x
0.75x
0.5x
Media

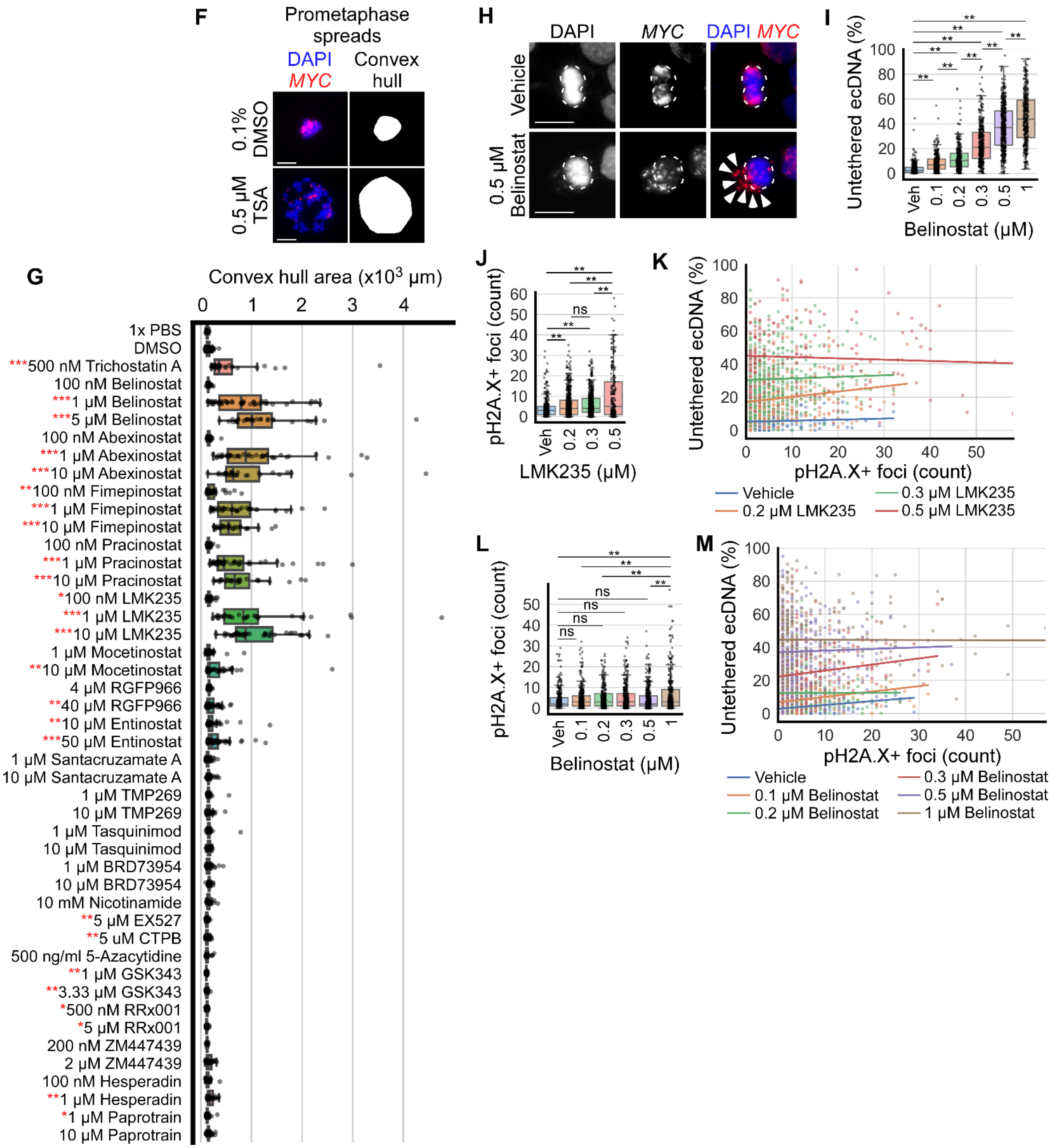

Figure S2. Related to Fig. 2.

A, metaphase COLO320DM cells treated for 15 min with 1x media, 0.75x media (not shown), 0.5x media, 1:1 mix of 1x PBS with 1x media (1x PBS-media, not shown), and 1:1 mix of 1.5x PBS with 1x media (1.25x PBS-media); dashed outlines indicate metaphase plate chromosomes, arrowheads indicate pH2A.X foci. ***B,*** boxplots quantifying the number of pH2A.X foci per metaphase cell from panel A; from left to right, n=3, 3, 3, 3, 3 biological

replicates and 304, 515, 576, 338, 506 cells; one-way ANOVA, F=5.4, p<.001, **p<.01 by Tukey's HSD, ns = not significant. ***C,*** correlation of the number of pH2A.X foci with ecDNA untethering for each treatment condition from panel B. ***D,*** COLO320DM cells arrested in metaphase with 10 μM MG132, treated for 3 hr with vehicle (regular media) or 10 mM NaN_3 and 50 mM 2-Deoxy-D-glucose (2-DG) dissolved directly in cell culture media and then incubated for 15 min in 1x media, 0.75x media (not shown), or 0.5x media; dashed outlines indicate metaphase plate chromosomes, arrowheads indicate untethered ecDNA. ***E,*** quantification of untethered ecDNA per cell from panel D; n=4, 4, 4, 4, 4, 4 biological replicates and 328, 194, 341, 174, 272, 169 cells; two-way ANOVA, NaN3 + 2DG: F=230.9, p<.001; media: F=657.8, p<.001; interaction: F=50.4, p<.001, **p<.01 by Tukey's HSD, ns = not significant. ***F,*** representative images of prometaphase spreads performed without incubation in hypotonic solution, performed on COLO320DM cells treated for 24 hr with 0.1% DMSO and 0.5 μM TSA treatment, along with the convex hull of the spread. ***G,*** boxplots quantifying the convex hull area of prometaphase spreads produced without incubation in hypotonic solution after 24 hr treatment with the indicated drug; from top to bottom, n=14, 205, 28, 49, 46, 30, 54, 40, 29, 44, 39, 29, 47, 44, 28, 39, 31, 43, 51, 55, 42, 55, 40, 59, 102, 114, 59, 38, 54, 31, 57, 54, 30, 60, 60, 18, 42, 43, 21, 24, 11, 31, 32, 21, 59, 62; *padj<0.05, **padj<0.001, ***padj<1e-10, padj = adjusted Student's t-test p value using Bonferroni multiple test correction (44 total comparisons were made). ***H,*** metaphase COLO320DM cells cultured on glass coverslips treated for 24 hr with vehicle (0.1% DMSO) or indicated concentrations of Belinostat; dashed outlines indicate metaphase plate chromosomes, arrowheads indicate untethered ecDNA. ***I,*** boxplots quantifying untethered ecDNA per cell from panel H; n=3, 3, 3, 3, 3, 3 biological replicates and 291, 310, 276, 357, 430, 316 cells; one-way ANOVA, F=424.3, p<.001, **p<.01 by Tukey's HSD. ***J,*** quantification of the number of pH2A.X foci per metaphase COLO320DM cell treated for 24 hr with vehicle (0.1% DMSO) or indicated concentration of LMK235; from left to right, n=5, 5, 4, 3 biological replicates and 331, 515, 472, 215 cells; one-way ANOVA, F=39.8, p<.001, **p<.01 by Tukey's HSD. ***K,*** correlation of the number of pH2A.X foci with ecDNA untethering for each treatment condition from panel J. ***L,*** quantification of the number of pH2A.X foci per metaphase COLO320DM cell treated for 24 hr with vehicle (0.1% DMSO) or indicated concentration of Belinostat; n=3, 3, 3, 3, 3, 3 biological replicates and 291, 310, 276, 357, 430, 316 cells; one-way ANOVA, F=6.2, p<.001, **p<.01 by Tukey's HSD. ***M,*** correlation of the number of pH2A.X foci with ecDNA untethering for each treatment condition from panel L. In all panels, scale bar = 10 μm and each data point in graphs represents one cell.

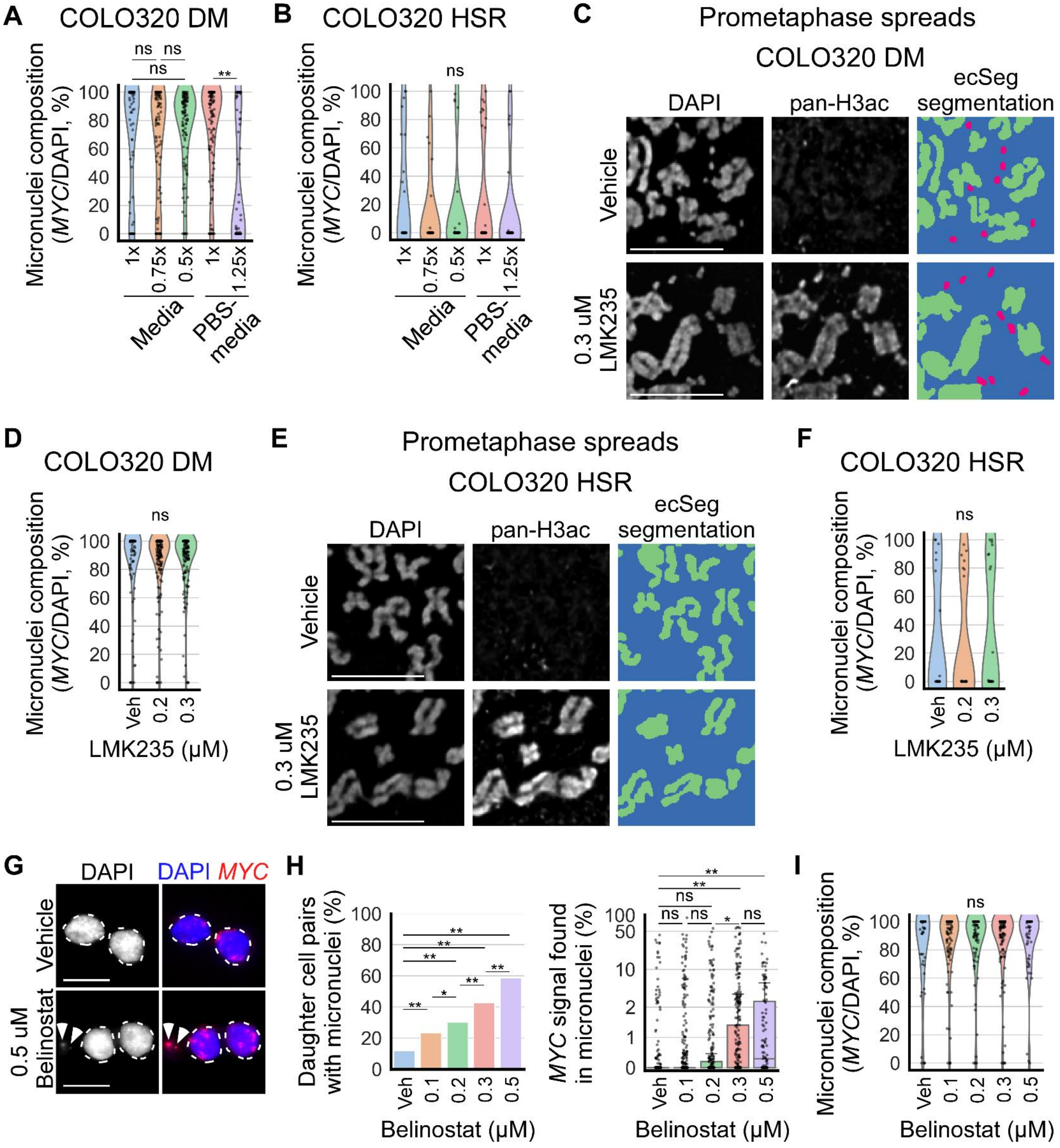

Figure S3. Related to Fig. 3.

***A*,** violin plots quantifying micronuclei composition (area of *MYC* FISH signal divided by area of DAPI signal within micronuclei) in COLO320DM cells treated for 6 hr with 1x media, 0.75x media, 0.5x media, 1x PBS-media, and 1.25x PBS-media from Fig. 3B; n=3, 3, 3, 6, 4 biological replicates and 63, 85, 110, 131, 65 daughter cell pairs (all micronuclei combined per cell pair); one-way ANOVA, F=19.1, p<.001, **p<.01. ***B*,** same quantification as panel A but for COLO320HSR cells from Fig. 3D; n=3, 3, 3, 5, 3 biological replicates and 23, 24, 28, 36, 32

daughter cell pairs; one-way ANOVA, F=0.6, p=.63. ***C,*** cytospin preparations of COLO320DM prometaphase spreads treated in vehicle or LMK235 for 24 hr, with IF staining for pan-Histone H3 acetylation (pan-H3ac). ecSeg was used to classify pixels as either chromosome (green) or ecDNA (red). Quantified in Fig. 3F. ***D,*** violin plots quantifying micronuclei composition in COLO320DM daughter cell pairs treated for 24 hr with vehicle (0.1% DMSO) or indicated concentration of LMK235 from Fig. 3G; n=5, 5, 4 biological replicates and 104, 187, 171 daughter cell pairs (all micronuclei combined per cell pair); one-way ANOVA, F=0.1, p=.86. ***E,*** same as panel C, for COLO320HSR cells; quantified in Fig. 3I. ***F,*** same quantification as panel D but for COLO320HSR cells from Fig. 3J; n=4, 3, 3 biological replicates and 19, 22, 21 daughter cell pairs; one-way ANOVA, F=0.2, p=.80. ***G,*** newly-divided daughter COLO320DM cells, as identified by the presence of Aurora B staining, treated for 24 hr with vehicle (0.1% DMSO) or indicated concentrations of Belinostat; dashed outlines indicate primary nuclei, arrowheads indicate micronuclei. ***H,*** quantification of panel G. Left: quantification of the percentage of daughter cell pairs with micronuclei; chi-squared, *p<.05, **p<.01. Right: quantification of the percentage of all *MYC* FISH signal per daughter cell pair inside micronuclei (symlog scale, linear ≤2, log >2); one-way ANOVA, F=8.0, p<.001, *p<.05, **p<.01; n=3, 3, 3, 3, 3 biological replicates and 482, 483, 358, 333, 121 daughter cell pairs. ***I,*** violin plots quantifying micronuclei composition from panel G; n=3, 3, 3, 3, 3 biological replicates and 54, 107, 104, 135, 68 daughter cell pairs; one-way ANOVA, F=0.6, p=.64. In all panels, scale bar = 10 μm and each data point in graphs represents one daughter cell pair.

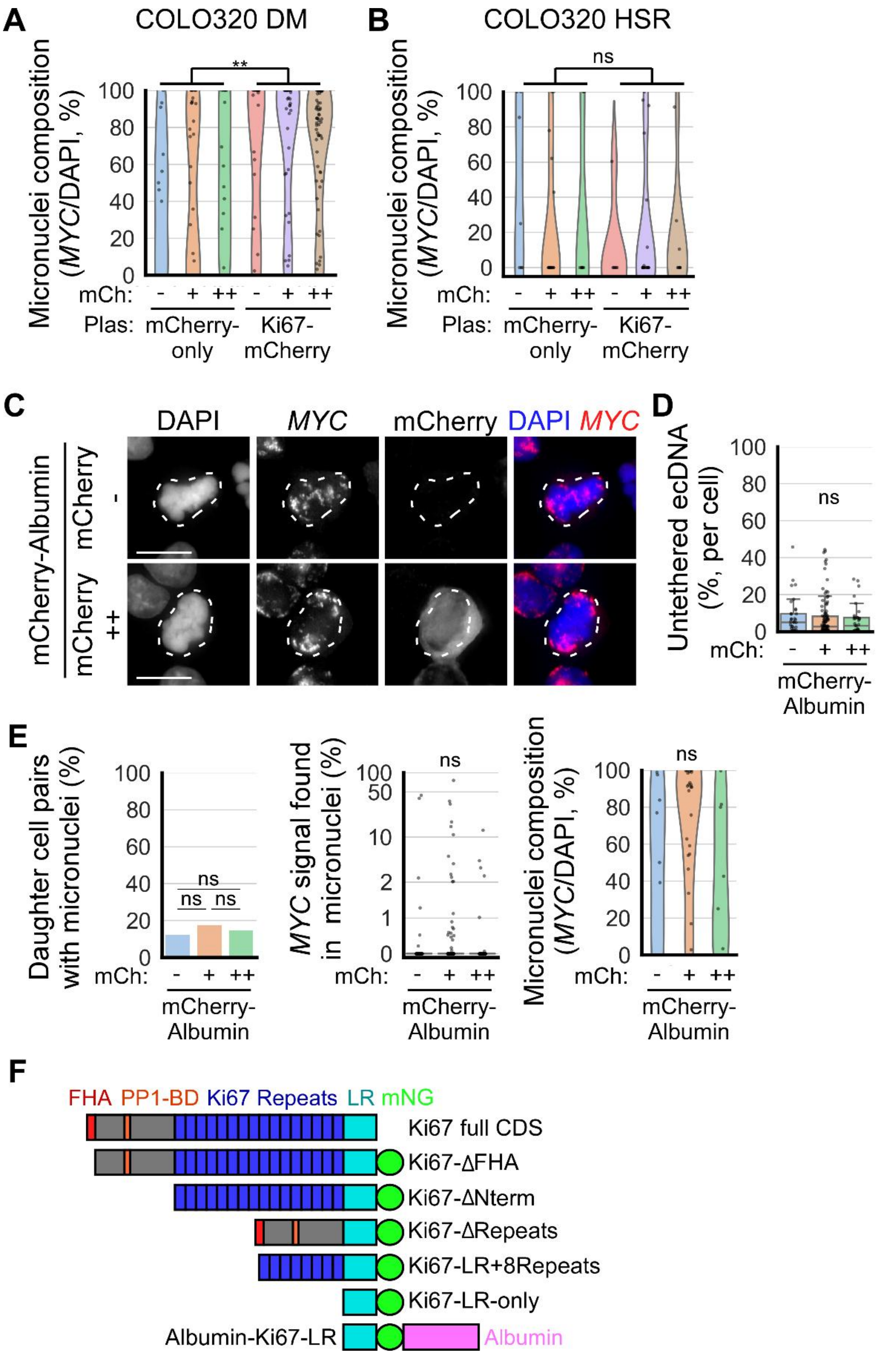

A
COLO320 DM
B
COLO320 HSR
Micronuclei composition (MYC/DAPI, %)
**
ns
mCh: - + ++ - + ++
Plas: mCherry-only Ki67-mCherry
C
DAPI
MYC
mCherry
DAPI MYC
mCherry-Albumin
mCherry -
mCherry ++
D
Untethered ecDNA (%, per cell)
ns
mCh: - + ++
mCherry-Albumin
E
Daughter cell pairs with micronuclei (%)
ns
MYC signal found in micronuclei (%)
Micronuclei composition (MYC/DAPI, %)
mCh: - + ++
mCherry-Albumin
F
FHA PP1-BD Ki67 Repeats LR mNG
Ki67 full CDS
Ki67-ΔFHA
Ki67-ΔNterm
Ki67-ΔRepeats
Ki67-LR+8Repeats
Ki67-LR-only
Albumin-Ki67-LR
Albumin

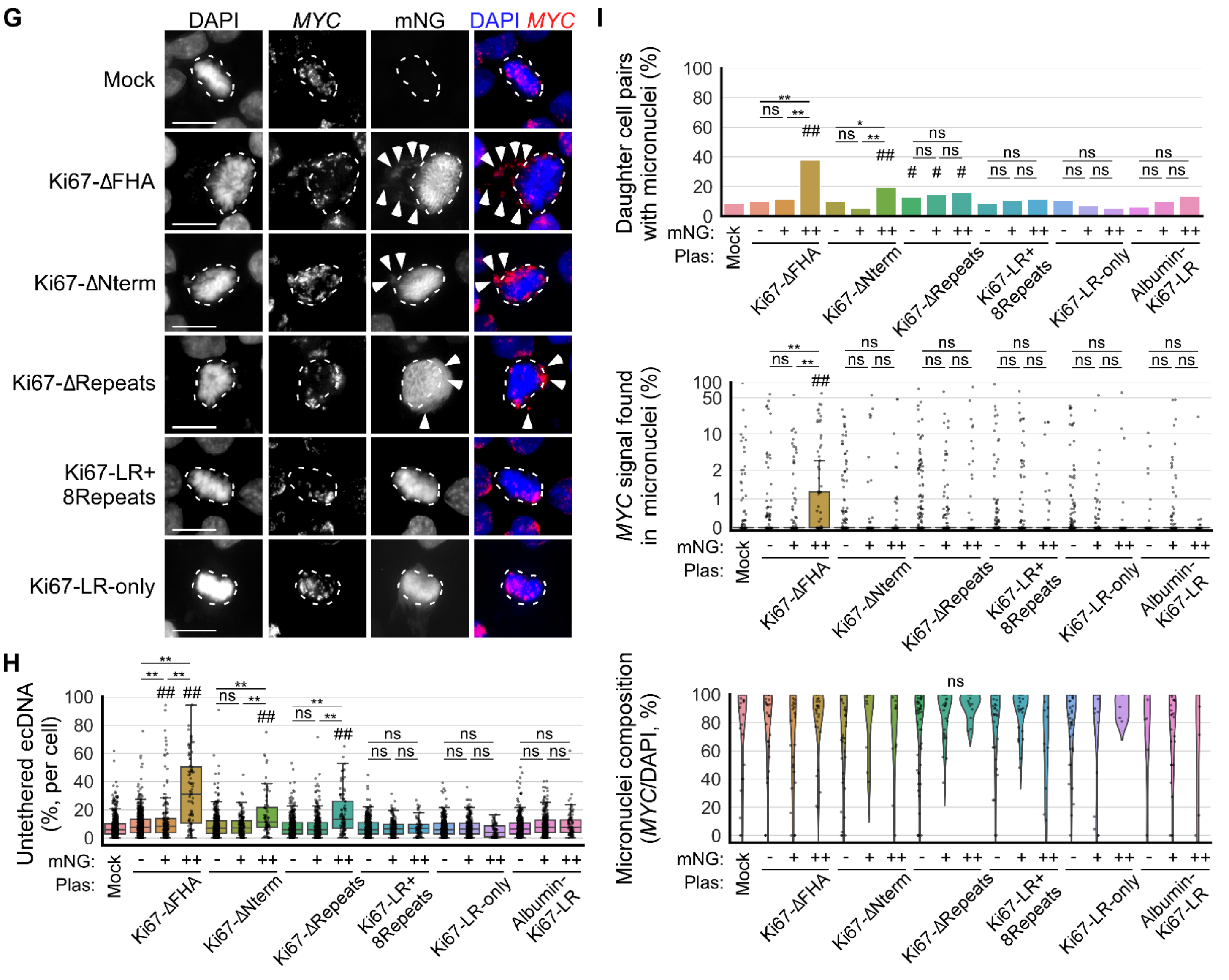

Figure S4. Related to Fig. 4.

A, violin plots quantifying micronuclei composition in COLO320DM daughter cell pairs 2 to 4 days post mCherry-only or Ki67-mCherry expression plasmid transfection from Fig. 4E; mCh = mCherry expression category, Plas = plasmid transfected; from left to right, n=7, 7, 7, 7, 7, 7 biological replicates and 13, 31, 15, 23, 53, 55 daughter cell pairs (all micronuclei combined per cell pair); two-way ANOVA, mCherry expression category (- vs + vs ++): F=0.4, p=.70; plasmid transfected (mCherry-only vs Ki67-mCherry): F=9.3, p=.0027; interaction: F=0.0, p=.97, ns = not significant. ***B,*** same quantification as panel A but for COLO320HSR cells from Fig. 4G; n=4, 4, 4, 4, 4, 4 biological replicates and 7, 18, 8, 6, 20, 8 daughter cell pairs; two-way ANOVA, mCherry expression category (- vs + vs ++): F=0.2, p=.83; plasmid transfected (mCherry-only vs Ki67-mCherry): F=0.9, p=.34; interaction: F=0.9, p=.40. ***C,*** metaphase COLO320DM cells 2 to 4 days post mCherry-Albumin (albumin coding sequence with signal peptide removed) expression plasmid transfection; cells were categorized based on mCherry fluorescence: - indicates lack of mCherry expression, ++ indicates top 20%tile mCherry expression by fluorescence intensity, + indicates the remaining cells that express mCherry; dashed outlines indicate chromosomes aligned at the metaphase plate. ***D,*** boxplots quantifying ecDNA untethering in panel C; n=3, 3, 3 biological replicates and 36, 106, 37 cells; one-way ANOVA, F=0.4, p=.70. ***E,*** left: quantification of the percentage of COLO320DM daughter cell pairs transfected with mCherry-Albumin plasmid with micronuclei, chi-squared. Middle: quantification of the percentage of all *MYC* FISH signal per daughter cell pair inside micronuclei, one-way ANOVA, F=0.5, p=.64; n=4, 4, 4 biological replicates and 71, 162, 60 daughter cell pairs. Right: quantification

of micronuclei composition, one-way ANOVA, F=1.9, p=.17; n=4, 4, 4 biological replicates and 9, 27, 8 daughter cell pairs. ***F,*** schematic of Ki67 domains and truncated Ki67 constructs (adapted from (49)), mNG = mNeonGreen. ***G,*** metaphase COLO320DM cells 2 to 4 days post transfection with truncated Ki67 constructs; dashed outlines indicate chromosomes aligned at the metaphase plate; arrowheads indicate untethered ecDNA. ***H,*** boxplots quantifying ecDNA untethering in panel G; cells were categorized based on mNeonGreen fluorescence: - indicates lack of mNeonGreen expression, ++ indicates top 10%tile mNeonGreen expression by fluorescence intensity, + indicates the remaining cells that express mNeonGreen; n=5, [5, 5, 5], [5, 5, 5], [5, 5, 5], [5, 5, 5], [5, 5, 5], [5, 5, 5] biological replicates and 555, [532, 168, 81], [414, 152, 66], [368, 386, 86], [400, 211, 71], [336, 268, 70], [385, 358, 85] cells; two-way ANOVA, mNeonGreen expression category (- vs + vs ++): F=120.8, p<.001, plasmid transfected (mock vs Ki67 truncation mutants): F=39.3, p<.001, interaction: F=34.4, p<.001, **p<.01 for indicated pair-wise comparisons, ##p<.01 compared to mock by Tukey's HSD. ***I,*** quantification of COLO320DM cells 2 to 4 days post transfection with truncated Ki67 constructs. Top: quantification of the percentage of COLO320DM daughter cell pairs with micronuclei, chi-squared, *p<.05, **p<.01, #p<.05 compared to mock, ##p<.01 compared to mock. Middle: quantification of the percentage of all *MYC* FISH signal per daughter cell pair inside micronuclei (symlog scale, linear ≤2, log >2), two-way ANOVA, mNeonGreen expression category (- vs + vs ++): F=15.2, p<.001, plasmid transfected (mock vs Ki67 truncation mutants): F=1.7, p=.12, interaction: F=3.5, p<.001; n=4, [4, 4, 4], [4, 4, 4], [4, 4, 4], [4, 4, 4], [4, 4, 4], [4, 4, 4] biological replicates and 502, [469, 351, 93], [572, 250, 93], [648, 259, 102], [673, 323, 113], [554, 412, 109], [193, 334, 60] daughter cell pairs. Bottom: quantification of micronuclei composition, two-way ANOVA, mNeonGreen expression category (- vs + vs ++): F=0.5, p=.50, plasmid transfected (mock vs Ki67 truncation mutants): F=1.4, p=.21, interaction: F=1.4, p=.18; n=4, [4, 4, 4], [4, 4, 4], [4, 4, 4], [4, 4, 4], [4, 4, 4], [4, 4, 4] biological replicates and 35, [39, 35, 33], [51, 12, 15], [65, 33, 14], [43, 31, 12], [51, 24, 5], [8, 30, 6] daughter cell pairs. In all panels, scale bar = 10 μm and each data point in graphs represents one cell or daughter cell pair.

A

gRNA #1

		Sequence	Mutation
Wildtype		TTACAGGAGCCAGCACGTCGTGTCTCAAGA	
Ki67 Knockout clone 1	allele 1	TTACAGGAGCCAGCACCGTCGTGTCTCAAGA	1 bp insertion
	allele 2	TTACAGGAGCCAGCAC........TCAAGA	8 bp deletion
clone 2		TTACAGGAGCCAGC.....GTGTCTCAAGA	5 bp deletion
clone 3		TTACAGGAGCCAGCA........GTGTGGAC<br>TCTGTTGCCATCTGCAGACCAAAGTCTTCTG<br>TGTCTGGTTAGAGGTAGGCAACCACTCAAGA	8 bp deletion,<br>63 bp insertion
clone 4		TTACAGGAGCCAGCAACGTCGTGTCTCAAGA	1 bp insertion
clone 5		TTACAG.............GTGTCTCAAGA	13 bp deletion
clone 6	allele 1	TTACAGGAGCCAGCAACGTCGTGTCTCAAGA	1 bp insertion
	allele 2	TTACAGGAGCCAGCACCGTCGTGTCTCAAGA	1 bp insertion

gRNA #2

		Sequence	Mutation
Wildtype		ACGAGACGCCTGGTTACTATCAAAAGGAGCGGGGTC	
Ki67 Knockout clone 1		ACGAGACGCCTGGTTAC....AAAAGGAGCGGGGTC	4 bp deletion
clone 2		ACGAGACGCCTGGTTACTATCCAAAAGGAGCGGGGTC	1 bp insertion
clone 3	allele 1	ACGAGACGCCTGGTTACTATCAAAGAGGAGCGGGGTC	1 bp insertion
	allele 2	ACGAGACGCCTGGTTACTA..AAAAGGAGCGGGGTC	2 bp deletion
clone 4	allele 1	ACGAGACGCCTGGTTACTAT.AAAAGGAGCGGGGTC	1 bp deletion
	allele 2	ACGAGACGCCTGGTTACTATCTAAAAGGAGCGGGGTC	1 bp insertion
clone 5	allele 1	ACGAGA......................GCGGGGTC	22 bp deletion
	allele 2	ACGAGACGCCTGGTTACTATCCAGATAAGCGGGGCCTGGGG	large insertion

B

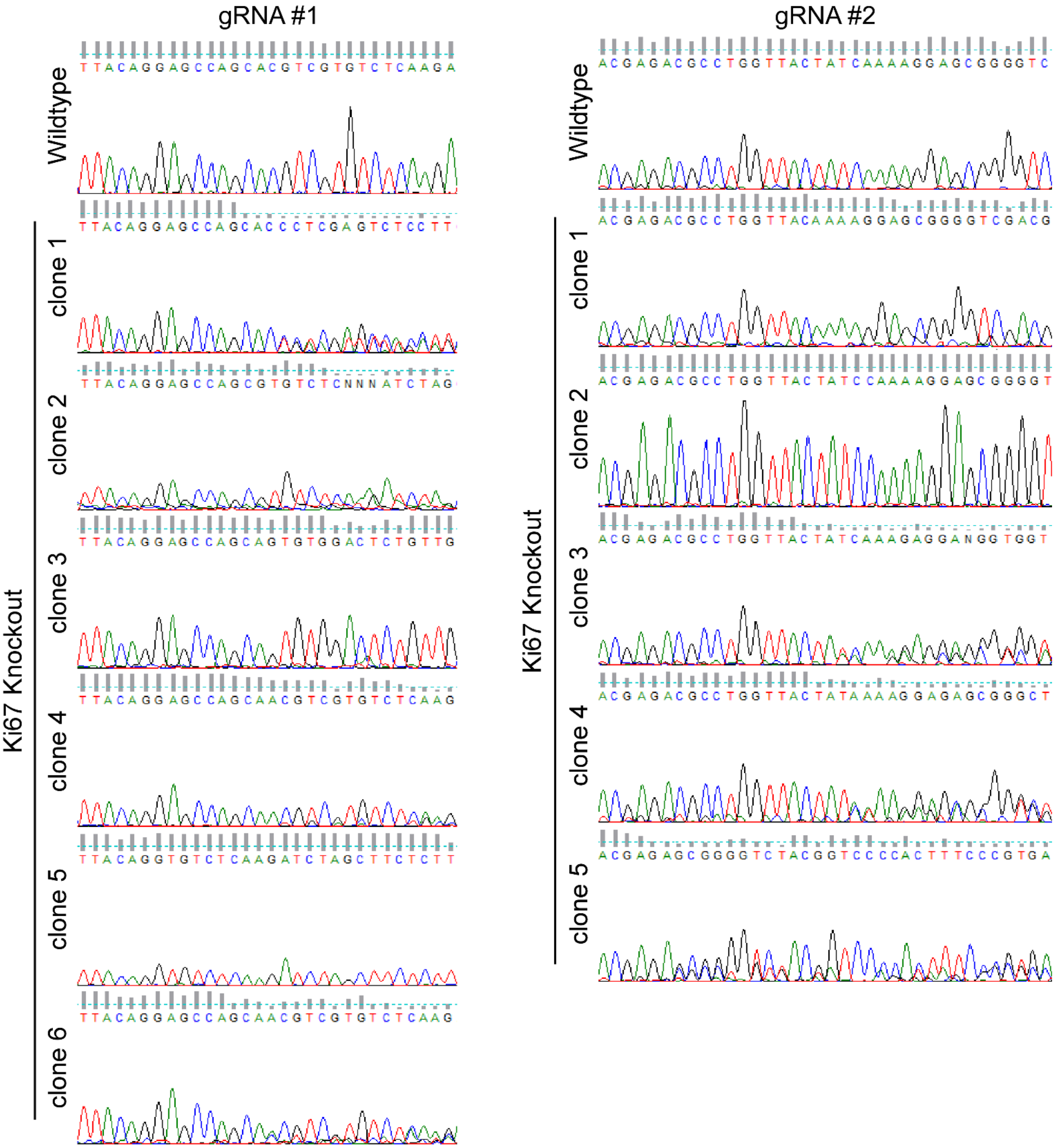

gRNA #1
gRNA #2
Wildtype
Ki67 Knockout
clone 1
clone 2
clone 3
clone 4
clone 5
clone 6

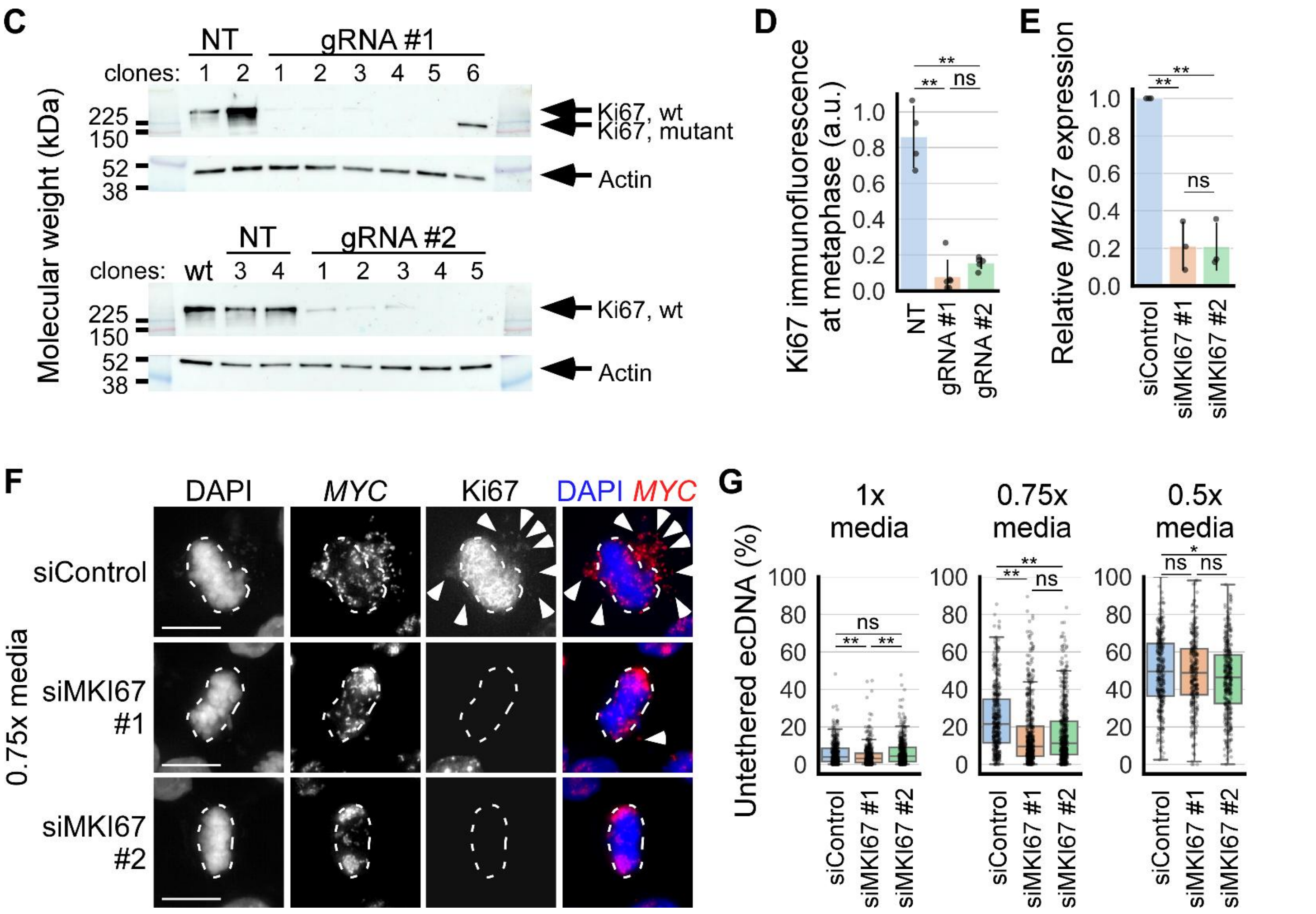

Figure S5. Related to Fig. 5.
A**,** indel mutations generated in each *MKI67* knockout clone by CRISPR-Cas9 mediated gene editing of *MKI67* at exon 6 (gRNA #1) and exon 2 (gRNA #2). ***B*****,** DNA sequence chromatograms at the CRISPR-Cas9 target site for each clone. ***C*****,** western blot performed on whole cell lysates of wildtype COLO320DM cells (wt, not transfected with Cas9) and *MKI67* wildtype and knockout clones generated using CRISPR-Cas9 mediated gene editing with non-targeting (NT) and *MKI67*-targeting gRNAs (gRNA #1 and #2). ***D*****,** quantification of Ki67 IF signal by integrated density in metaphase cells of COLO320DM wildtype (NT) and *MKI67* knockout clones (gRNA #1 and #2); each data point represents the average of at least 122 cells from each clone; from left to right, n=4, 6, 5 clones; one-way ANOVA, F=70.1, p<.001, **p<.01 by Tukey's HSD, ns = not significant; error bars = mean ± standard deviation; a.u. = arbitrary units. ***E*****,** quantification of *MKI67* mRNA expression in COLO320DM cells two days post transfection with non-targeting siRNA (siCtrl) or one of two siRNAs targeting *MKI67* (siMKI67 #1 and #2); qRT-PCR, $2^{-\Delta\Delta CT}$ analysis (normalized to *GAPDH* and siCtrl); n=3, 3, 3 biological replicates; one-way ANOVA, F=56.2, p<.001, **p<.01 by Tukey's HSD, ns = not significant; error bars = mean ± standard deviation. Each data point represents one biological replicate. ***F*****,** metaphase COLO320DM cells cultured on glass coverslips two days post siRNA transfection, incubated for 15 min in 1x media (not shown), 0.75x media, and 0.5x media (not shown); dashed outlines indicate chromosomes aligned at the metaphase plate, arrowheads indicate untethered ecDNA; scale bar = 10 µm. ***G*****,** boxplots quantifying ecDNA untethering in siRNA transfected metaphase COLO320DM cells from panel F after 15 min incubation in 1x media (left; n=3, 3, 3 biological replicates and 419, 454, 477 cells; one-way ANOVA, F=56.2, p<.001), 0.75x media (middle; n=3, 3, 3 biological replicates and 407, 572, 543 cells; one-way ANOVA, F=41.0, p<.001), and 0.5x media (right; n=3, 3, 3 biological replicates and 336, 313, 366 cells; one-way ANOVA, F=3.9, p=.02); *p<.05, **p<.01 by Tukey's HSD. Each data point represents one cell.
